# Behaviour change techniques in brief interventions to prevent HIV, STI and unintended pregnancies: A systematic review

**DOI:** 10.1371/journal.pone.0204088

**Published:** 2018-09-27

**Authors:** Sofia De Vasconcelos, Igor Toskin, Bergen Cooper, Marie Chollier, Rob Stephenson, Karel Blondeel, Thierry Troussier, James Kiarie

**Affiliations:** 1 Department of Reproductive Health and Research, World Health Organization, Geneva, Switzerland; 2 Center for Health and Gender Equity (CHANGE), Washington, D.C., United States of America; 3 UNESCO Chair for Sexual Health and Human Rights (UCSHHR), Paris, France; 4 Manchester Metropolitan University, Manchester, United Kingdom; 5 School of Nursing and the Center for Sexuality and Health Disparities, University of Michigan, Ann Arbor, Michigan, United States of America; 6 Faculty of Medicine and Health Sciences, Ghent University, Ghent, Belgium; Indiana University School of Medicine, UNITED STATES

## Abstract

**Background:**

Behaviour-change interventions have been consistently considered an essential part of comprehensive HIV, STI and unintended pregnancy prevention. In 2015, the World Health Organization reviewed and assessed existing evidence on brief behavioural interventions, leading to the publication of *Brief sexuality-related communication*: *recommendations for a public health approach*. This guideline recommends the use of brief behaviour intervention and communication programmes to promote sexual health and to prevent HIV, STIs, and unintended pregnancies in primary health services, particularly sexual and reproductive health services.

**Objective:**

With the purpose of informing the development of a brief behaviour intervention in sexual and reproductive health, we conducted a systematic review of brief intervention to prevent HIV, STI and unintended pregnancies, to identify behaviour change techniques (BCTs) used in health care settings.

**Methods:**

Participants from all ages and genders were included. Brief interventions delivered in ≤ 60 minutes were included. Data was extracted, and interventions were coded following the Behaviour Change Techniques Taxonomy (BCTTv1) guidelines.

**Results:**

Of the 6.687 articles identified, 355 were reviewed and 37 studies were included. In effective interventions, we identified 48 behaviour change techniques (BCTs). A core set of 8 frequently used behaviour change techniques was identified: *“Problem solving”*, “*Feedback on behaviour”*, “*Social support (unspecified)”*, *“Instructions on how to perform the behaviour”*, *“Information about health consequences”*, *“Information about social and environmental consequences”*, *“Demonstration of the behaviour”* and *“Credible source”*.

**Conclusions:**

The technical content of brief behaviour interventions was identified in a reliable and standardized way providing preliminary indications on potentially effective techniques to achieve behaviour change.

## Introduction

Sexual health is a state of physical, mental and social well-being in relation to sexuality [1, 2]. Sexual health encompasses the rights of all persons to have the knowledge and the opportunity to pursue a safe and threat-free sexual life [3]. Sexual health is a broad area encompassing many inter-related challenges and problems related to sexual and gender identity, sexual expression, relationships, pleasure as well as negative consequences or conditions such as infections with the human immunodeficiency virus (HIV) and other sexually transmitted infections (STIs) and unintended pregnancies.

The ability of people to achieve positive sexual health depends on, among other things, their access to comprehensive information about sexuality, their knowledge about sexual risks, their vulnerability to the adverse consequences of sexual activity and on their access to good-quality sexual health care; which includes brief behaviour interventions and communication programmes [1, 4]. In 2015 the World Health Organization (WHO) Department of Reproductive Health and Research (RHR) published *Brief sexuality-related communication guidelines*: *recommendations for a public health approach* (BSC guidelines). This technical advice on the improvement of sexual health within primary care recommends the use of “counselling skills opportunistically (…) to address sexuality and related personal or psychological problems”, to promote sexual health and to prevent STIs (including HIV) among adults and adolescents in primary health services [1, 4]. Subsequent to this recommendation, the *Brief Sexuality-related Communication (BSC)* intervention will be developed. This brief intervention aims to identify current and potential sexual health problems and motivate those at risk to change their sexual behaviour or maintain safer sexual behaviours to achieve identified sexual health goals. Based on the principles of attendance, response, personalization and initiation, the *BSC* intervention will encompass proven behaviour change techniques to address issues related to following risks that arise from unsafe sexual practices: infection with HIV or other sexually transmitted infections (STIs) and unintended pregnancies [1, 3].

Recent systematic reviews [4, 5, 6] and meta-analyses [7, 8] showed the effectiveness of brief behavioural interventions to prevent HIV and STI transmission. Advances in behavioural science have generated methods for furthering identifying the techniques used in these interventions. Among these methods, the Behaviour Change Techniques Taxonomy version 1 (BCTTv1) developed by Michie et al. [9, 10, 11], is a comprehensive and useful framework to report in a standardised and reliable way the technical components of interventions, the Behaviour Change Techniques (BCTs). By technique, Michie et al. refer to an observable, replicable component of an intervention designed to alter or redirect causal processes that regulate behaviour; in other words, a technique is viewed by the authors as an “active ingredient” of an intervention [9, 12]. This discrete and irreducible component of an intervention, that on its own has the potential to change behaviour, can be used (delivered) alone or in combination with other BCTs in behaviour change interventions [9, 13–16]. To address the gap in the literature regarding the specific techniques that are likely to bring about the effectiveness of interventions and inform the *Brief sexuality-related Communication (BSC)* intervention’s design, this paper reviews brief and effective interventions to prevent HIV, STI and unintended pregnancies. Interventions are analysed following the BCTTv1 in order to address the following research question: “Which behaviour change techniques (BCTs) are frequently identified as effective in brief interventions aimed at preventing outcomes around HIV, STIs and unintended pregnancies?”

## Methods

The methods involved a systematic review of studies with evidence of effectiveness of brief interventions for shifting sexual health related outcomes.

### Inclusion criteria

#### Types of studies

Randomized control trials (RCTs), observational studies, based in grounded theory, ethnography, descriptive, and phenomenological theory, were considered for inclusion. Discussion papers and commentaries were excluded.

#### Types of participants

Participants of all ages, genders and sexual orientations were included, whether sexually active or not.

#### Types of interventions

To be considered for inclusion, interventions needed to fit the criteria of BSC as defined by the WHO [1]. Interventions could have occurred face-to-face, or using multimedia, or with a combination of face-to-face and multimedia. Interventions included had to be delivered in one session of 60 minutes maximum, with the possibility of a booster session. Interventions had to be delivered in primary health care settings. Population level interventions (i.e. mass media campaigns, school-based sex education programs) were excluded.

#### Types of outcomes

Studies were included with sexual health-related biological and/or behavioural outcomes (i.e. HIV, STI and unintended pregnancies incidence and condom use). Follow-up had to have occurred at least 1 month after the intervention.

#### Electronic searches

We searched for MeSH and keywords terms on PubMed. Keyword searches on Summons (limited to ProQuest, CINAHL, Jstor, Scopus/Science Direct, Cochrane Library, EBSCO, CINAHL, Ovid Medline/PubMed, PsycInfo, Web of Knowledge) were performed crossing the following terms: brief therapy, brief communication, attending, empathy, client focus, observation, representation, open questions, active listening, emotional reformulation, motivation, personal goal, helping model, problem solving, empowerment, awareness, sexual health, reproductive health, primary care, cancer, sexual dysfunction, sexual distress, sexual concerns, sexual difficulties, sexual problems, sexual misconceptions, sexually transmitted infections, HIV, unwanted pregnancy, abortion, sexual violence, harmful practices, knowledge increase, well-being, autonomy, pleasure, training. There was no restriction on language. Studies had to be published between 1988 and 2014.

Further to the initial systematic review, a termly documentary monitoring, based on the same electronic search criteria, allowed the identification and potential inclusion of new studies published up to September 2017.

#### Other resources

In addition to the above described search strategy, both published and unpublished articles were also solicited from experts in the field of behaviour science as well as sexual and reproductive health through the WHO serve list. Some documents were identified through hand searching the references of included articles. Supporting materials (videos, leaflets, posters, training manuals) were consulted and used to code interventions when available.

### Data collection and analysis

#### Selection of studies

Following the PRISMA guidelines, abstracts were assessed for inclusion by two reviewers (SDV, BC) [17]. When matching inclusion criteria, full text articles were read by three reviewers (SDV, BC, IT).

#### Data extraction and management

Data was extracted by two reviewers (SDV, BC) and subsequently reviewed by a third reviewer (IT). Risk of bias was assessed for each study by one reviewer (BC). Two reviewers (SDV, MC) independently coded descriptions of intervention content into BCTs. Due to the heterogeneity in target populations, delivery methods, types of outcomes, a meta-analysis was not considered appropriate. Therefore results are synthetized in a systematic descriptive form.

#### Assessment of risk of bias in inclusion

Data were processed using REVMAN 5 software for quantitative data. Using REVMAN, bias assessment included random sequence, allocation, blinding of participants and personnel, blinding of outcome, incomplete data, selective reporting, as well as other potential sources of bias. One author entered the information into REVMAN and two authors checked for accuracy.

#### Characteristics of the studies and interventions

Data extraction of general study characteristics included study design, location of the study, population, demographic characteristics of the population, sample size, primary outcomes as well as length of follow-ups. The characteristics of the interventions, such as the name of the intervention, the theoretical frameworks were extracted. Regarding the procedures for the delivery of the intervention, the settings, the modes of delivery, intervention fidelity, as well as the level, the intensity and the duration of the intervention were collected.

#### BCTs coding and analysis

The 93-item BCTTv1 coding frame developed by Michie et al. is an extensive and consensually agreed taxonomy of BCTs, providing standardized definitions and labels of each behaviour change technique. This taxonomy is hierarchically organized into 16 clusters: 1) Goals and planning; 2) Feedback and monitoring; 3) Social support; 4) Shaping knowledge; 5) Natural consequences; 6) Comparison of behaviour; 7) Associations; 8) Repetition and substitution; 9) Comparison of outcomes; 10) Reward and threat; 11) Regulation; 12) Antecedents; 13) Identity; 14) Scheduled consequences; 15) Self-belief; and 16) Covert learning [12]. The taxonomy also includes detailed coding instructions allowing to identify and accurately describe technical intervention components that bring about behaviour change in a standardized way across heterogeneous and often complex behaviour change interventions. According to the taxonomy authors, BCTTv1 is a reliable method for extracting information about interventions content, and identifying and synthetizing discrete and replicable, potentially active ingredients (or combination of ingredients) associated with effectiveness [9]. The BCTTv1 has been used in different behavioural domains such as physical activity, smoking cessation, healthy eating, alcohol use, changing professional behaviour and condom use [16, 18–21]. Two psychologists followed the online training programme provided by the authors of the BCTTv1 (http://www.bct-taxonomy.com/) and then coded independently the technical content of experimental and control conditions following the standard guidelines [9, 13–15]. BCTs were identified within published studies, and when available, within supplementary materials related to the interventions (i.e. manuals for use, manuals for training, guides for technical assistance, protocols, texts describing interventions and printed materials such as posters and brochures). According to the BCTTv1 taxonomy developers, coding task’s disagreements were resolved by consensus through discussion [22, 23]. The number of BCTs identified in interventions as well as the number of effective and control/ineffective interventions incorporating each technique was calculated and assessed. Regarding the frequency with which a technique was used in effective interventions, following Michie et al. [11, 16,21], we considered a BCT as frequent when the technique was identified in at least 50% of the interventions. Additionally, interventions were also assessed according to their intensity (1 session vs 1 + 1 session interventions), their duration (≤ 40 min vs >40 min interventions), their mode of delivery (health provider-delivered vs health provider and multimedia-delivered interventions), and the unit of the intervention (individual vs group interventions). Weighted Cohen’s Kappa was calculated to assess the reliability of the independent coding and to determine the level of agreement between the two coders on the presence or absence of BCTs in the interventions.

#### Target behaviour of BCTs

In this study, the target of BCTs identified with the BCTTv1 was condom use (to prevent HIV, STI and/or unintended pregnancies). When interventions encompassed several target behaviours, only the BCTs related to the relevant target behaviour were coded.

#### “Standard of care”, “standard counselling” and control group conditions

When described and related to the relevant target behaviour, control groups (“standard of care”, “HIV counselling”, “standard HIV counselling”) were coded following the BCTTv1 coding guidelines.

#### Coding assumptions

In the absence of further details but with precision about the target behaviour (condom use), “standard of care” groups were coded with two BCTs: *3*.*1 “Social support (unspecified)”* and *9*.*1 “Credible source”* when delivered by a health provider, a clinician or a nurse. Regarding “HIV prevention counselling”, the following BCTs were coded: *3*.*1 “Social support (unspecified)”*, *9*.*1 “Credible source”*, *5*.*1 “Information about health consequences”* when no further details of the intervention content were provided. Regarding printed materials (i.e. brochures, posters and pamphlets) targeting the relevant behaviour, and involving a provision of HIV, STI and unintended pregnancies “education” or “information”, without any further details, we assumed that they could be coded at minimum: *5*.*1 “Information about health consequences”* and *4*.*1 “Instructions on how to perform the behaviour”*.

## Results

### Articles retrieved

**Fig 1:**
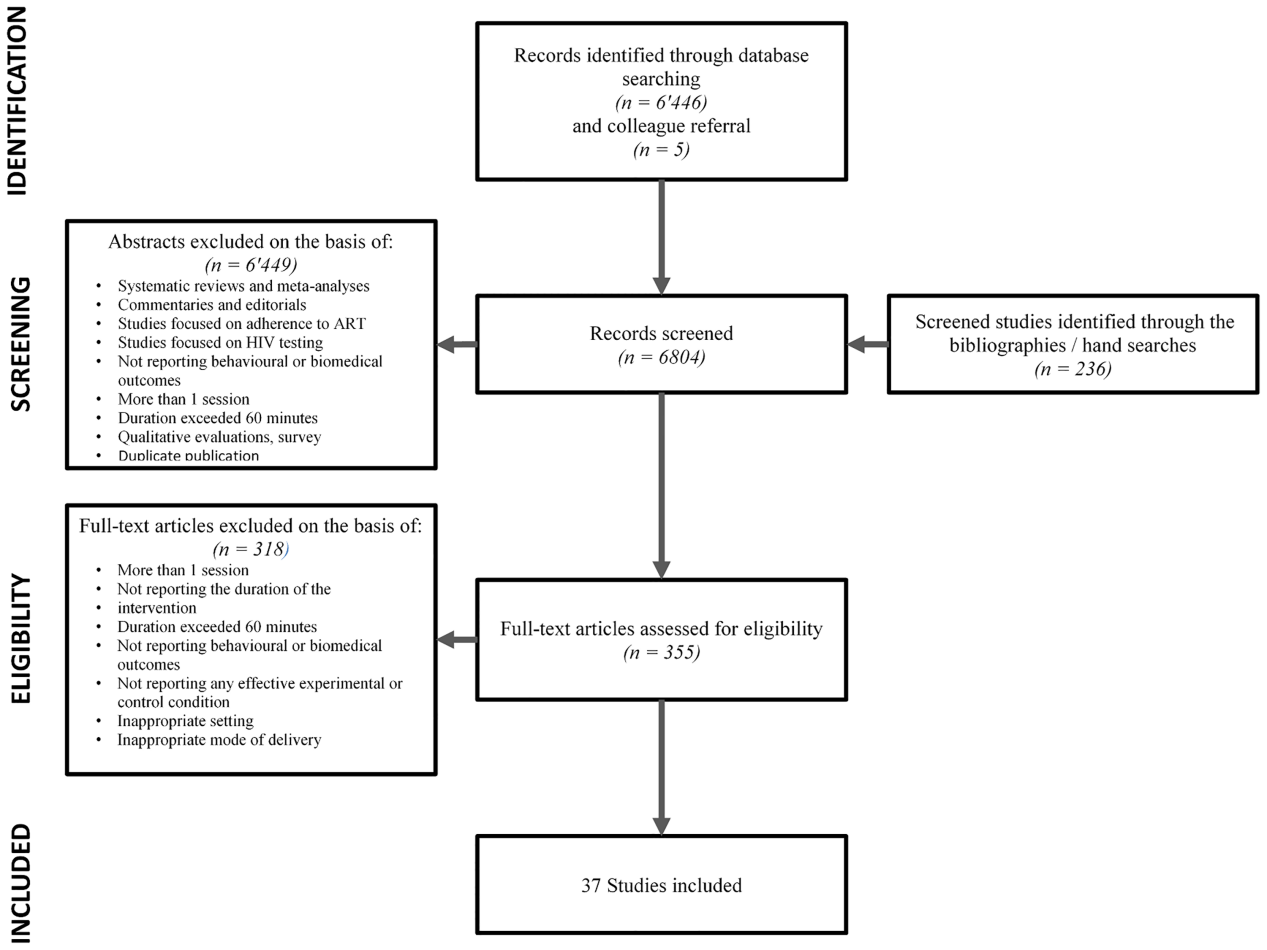
Flowchart.

After reviewing abstracts for inclusion, 355 full text articles were assessed for inclusion. We excluded 231 articles in which interventions were delivered in more than one session or/and because their intervention’s duration exceeded 60 minutes, or the duration was not mentioned. Eighty-seven additional studies were excluded based on not matching inclusion criteria as type of outcome, mode of delivery, setting, and/or based on their ineffectiveness in preventing HIV, STI and unintended pregnancies. In total, we included 37 studies equalling 56536 participants. A flowchart of included studies can be found in Fig 1.

### General characteristics of the studies and interventions

Papers were published between 1992 and 2017. Table 1 describes the main characteristics of the included studies. Most studies were RCTs (n = 28), quasi-experimental studies (n = 4) [24–27], secondary analyses of RCTs (n = 2) [28, 29] and three had other experimental study designs (n = 3) [30–32]. The majority of the studies were conducted in the USA (including Puerto Rico) (67.5%) and South Africa (13.5%) [29, 33–36]. The remaining took place in Spain [18, 37], Mexico [38, 39], Russia [40], the Netherlands [26] and Uganda [32]. Regarding the type of interventions, 18 studies assessed the effectiveness of HIV prevention interventions [18, 24–28, 32–37, 40–46], and 19 assessed HIV and STI prevention interventions [29, 31, 35, 38, 39, 47–49], STI prevention interventions [30, 50–57], and STI and unintended pregnancies prevention interventions [58, 59]. Thirty-four studies took place in primary health care settings defined as: STI clinic, HIV clinic, Teen Health Clinic, Municipal Public Health Clinic, Paediatrician Clinic, Family planning, Medical Clinic, HIV testing site, Community Health Center, or Outpatient Clinic. In three studies, the settings were a University clinical psychology laboratory and an HIV/AIDS prevention, treatment and research unit [18, 37, 43]. In one study the settings were temporary service sites services for homeless women [27].

**Table 1:** General characteristics of the studies.

Reference	Setting/country	Population and sample size	(1) Study design (2) Experimental condition(s) (3) Control condition	Intervention features		Follow-up	(1) Behavioural outcomes (2) Biomedical outcomes
				Theoretical framework	(1) Delivery method (2) Intervention level (3) Intensity (4) Duration (5) Fidelity		
**Abdala et al. 2013** [40]	STD clinic, Russia	STI clinic patients71,66% men, 28.3% women, mean age: 26.9 yrs, Sample size: 307	(1) Randomized controlled trial (2) HIV counselling (3) Written HIV information	IMB	(1) Health provider (2) Individual (3) 1 session (4) 60 min (5) Training + flip chart was used to deliver the intervention + debriefing sessions+ multicultural factors and literacy issues addressed	3, 6 months	(1) Intervention significantly increased percentage of condom use, consistent condom uses and significantly decreased of unprotected sexual acts at 3-months follow-up.
**Artz et al. 2005** [58]	STD clinic, USA	STI clinic patients, Women STI Clinics’ patients, 88% Black, 11,4% White, mean age: 25 yrs, Sample size: 427	(1) Randomized controlled trial (2) Enhanced intervention (3) Basic intervention	Not reported	(1) Health provider + video + printed materials (2) Individual (3) 1 session + 1 boost session (4) 49 min + 20 min (boost session) (5) Training to deliver the scripted intervention + flipchart used to deliver the intervention + training session with video role-paying sessions and feedback	2, 6 months	(1) The enhanced intervention increased use of condoms and vaginal microbicide at 6-month follow-up. (2) No significant difference in rates of STD cases between intervention groups. No proportionate reduction in the rate of pregnancies in the enhanced intervention group as compared to the basic intervention group.
**Ballester-Arnal et al. 2014** [37]	Universitat Jaume 1, laboratory setting, Spain	Adolescents and young people, Spanish young people, 75% female, 90,08% heterosexual; mean age: 20,9 yrs; Sample size: 239	(1) Randomized controlled trial (2) “Talk” intervention, or “Website” intervention, or “Attitudinal discussion” intervention, or “Seropositive participant” intervention, or “fear induction” intervention, or “Role-play” intervention (3) No intervention	IMB	(1) Health provider; health provider + web; health provider + video + printed materials (2) Group (3) 1 session (4) 60 min (5) Cultural factors addressed	4 months	(1) Higher increase of safe sexual practices (including condom use) for participants of all conditions compared to the non-intervention. “Attitudinal discussion” and “Seropositive participant” have revealed the best improvement, at 4-months follow-up.
**Boekeloo et al. 1999** [59]	Clinical (paediatrician), USA	Adolescents and young people; 50,8 males; 49,2 females, 63% African American, age range: 12–15 yrs; Sample size: 219	(1) Random intervention Trial (2) “Assess” intervention (3) Standard-of-care	Social-Cognitive Theory; Theory of Reasoned Action	(1) Health provider + audiotape + printed materials (2) Individual (3) 1 session (4) 14 min (5) Training + exit interview after the intervention and at 3 and 9 months-follow-ups	3–9 months	(1) Among intervention’s participants, more sexually active adolescents reported condom use at 3- months’ follow-up. (2) More signs of STD were reported by control group than intervention group, at 9-months’ follow-up. No differences in reported pregnancies between control and intervention at 9-months follow-up.
**Carey et al. 2014** [48]	STD clinic, USA	STI clinic patients; 56% males, 44% females, 69% African American, mean age: 28.5 yrs; Sample size:1010	(1) Randomized controlled trial (2) General health survey and general health DVD, or general health survey and “Be the Change” sexual health DVD, or sexual health survey and general health DVD, or sexual health survey and “Be the Change” sexual health DVD	IMB	(1) Computer + video (2) Individual (3) 1 session (4) 22 min (5) Satisfaction with the intervention survey + multicultural factors addressed	3, 6, 9, 12 months	(1) Reduction of self-reported sexual risk behaviour in all conditions, at 3-, 6-, 9- and 12-months follow-ups. (2) Reduction of the incidence of new STDs in all conditions at 3-, 6-, 9- and 12-months follow-up.
**Cohen et al. 1992** [50]	STD clinic, USA	STI clinic patients; 70,9% men; 89,1% African American, mean age: 28,4 yrs; Sample size: 551	(1) Randomized controlled trial (2) Intervention (3) No intervention	Not reported	(1) Health providers + video (2) Group (3) 1 session (4) 45 min (5) Multicultural factors addressed	7, 9 months	(2) Lower rate of reinfection among participants of the intervention, at 7- to 9- months follow-up.
**Cohen et al. 1992** [51]	5 Public Health STD clinics, USA	STI clinic patients; 61,2% men, 38,7% women, 71,5% African American, mean age: 27,9 yrs (men) and 26,6 yrs (women); Sample size: 903	(1) Randomized controlled trial (2) “Skills approach” intervention, or “Social influences” intervention, or “Distribution” intervention (3) No intervention	Not reported	(1) Health providers (2) Group (3) 1 session (4) 15–20 min (5) Not reported	6, 9 months	(2) Fewer known reinfections for male participants of “Condoms skills” and “Social influences” interventions compared to male control subjects, at 6- to 9-months follow-ups. Increase of rates of infection for women participants of the “Social influences” intervention when compared to controls.
**Cornman et al. 2008** [33]	HIV care clinic South Africa	PLWH; 57% women, 43% male, 91% Zulu; mean age: 34 yrs; Sample size: 152	(1) Randomized controlled trial (2) “Izindlela/Zokuphila/Options for Health” intervention (3) Standard-of care HIV counselling	IMB; Motivational Interviewing principles	(1) Health provider (2) Individual (3) 2,5 sessions in average (regular scheduled medical visits) (4) 15 min (5) Training + “in vivo” observations + weekly supervision + multicultural factors and literacy issues addressed	6 months	(1) Significant decrease of the mean number of reported unprotected vaginal and anal events over the time among patients who received the intervention, at 6- months follow-up.
**Crosby et al. 2009** [52]	STD clinic, USA	STI clinic patients; 100% men, African American, mean age: 23,25 yrs; Sample size: 266	(1) Randomized controlled trial (2) HIV prevention intervention (3) Standard -of-care nurse-delivered STD prevention messages	IMB; Social Learning Theory	(1) Health provider + printed materials (2) Individual (3) 1 session (4) 45–50 min (5) Training + multicultural factors addressed	3, 6 months	(1) Increased report of condom uses during the last sexual intercourse for patients who received the intervention, at 6 months follow-up. (2) Patients who received the intervention were significantly less likely to acquire subsequent STDs, at 6-months follow-up.
**Crosby et al. 2014** [57]	STD clinics, USA	STI clinic patients; 100% men, African American, mean age: 19.6 yrs; Sample size: 702	(1) Randomized controlled trial (2) “Focus on the Future” (FoF) intervention (3) Attention-equivalent control condition	IMB; Social Learning Theory	(1) Health provider + printed materials (2) Individual (3) 1 session (4) 54.1 min (5) Training + tape-record of every 10^th^ intervention + multicultural factors addressed	2, 6 months	(1) Efficacy of the intervention for correct use of condom, at 6-months follow-up. (2) No significant effect on chlamydia and gonorrhea incidence.
**Crosby et al. 2017** [49]	2 STI clinics, USA	MSM; 100% HIV-positive and HIV-negative young black men who have sex with men (YBMSM), mean age: 22.6 yrs; Sample size: 600	(1) Randomized controlled trial (2) “Focus on the Future” (FoF) intervention (3) Standard-of care condom and lubricant supplies without teaching opportunity	IMB; Social Learning Theory	(1) Health provider + computer (2) Individual (3) 1 session (4) 45–50 min (5) Multicultural factors and literacy issues addressed	3, 6, 9, 12 months	(1) Efficacy of the intervention to decline the frequency of condomless anal receptive intercourse, for HIV -uninfected and HIV infected men, at 12-months follow-up (2) No effect
**Dilley et al. 2002** [46]	HIV- testing clinic, USA	MSM; 100% MSM, median age: 33 yrs; Sample size: 456	(1) Randomized controlled trial (2) Standard counselling + diary + HIV test, or intervention counselling + HIV test, or intervention counselling + standard HIV counselling + diary + HIV test (3) Standard HIV testing counselling + HIV test	Gold’s Model of “on-line” vs “off-line” self-appraisal of risk behaviour	(1) Health provider (2) Individual (3) 1 session (4) 60 min (5) Training + supervision + transcription and review of audiotaped interventions + satisfaction with the intervention questionnaire	6–12 months	(1) The counselling session significantly decreased the proportion of participants reporting UAI with non-steady partners of unknown or discordant HIV serostatus, at 6- and 12- months as compared with a control group when added to standard client-centred HIV counselling and testing.
**Dilley et al. 2007** [41]	HIV testing site, USA	MSM; 100% HIV negative men, 63% White, 11,8% Latino, age range: 19–30 yrs (29,55%), 31–40 yrs(46,6%), 41–60 yrs (23,8%); Sample size: 336	(1) Randomized controlled trial (2) “PCC” (Personalized Cognitive Counselling) intervention + HIV test (3) Standard HIV testing counselling + HIV test	Gold’s Model of “on-line” vs “off-line” self-appraisal of risk behaviour; Model of Relapse Prevention	(1) Health provider (2) Individual (3) 1 session (4) 50 min (5) Training + supervision + review of audiotaped sessions + satisfaction with the intervention questionnaire	6–12 months	(1) Men receiving the PCC intervention reported significantly less UAI with non-steady partners of non-concordant serostatus than those receiving the usual counselling, at 6- and 12-months follow-ups.
**Dilley et al. 2010** [28]	HIV testing site, USA	MSM; 100% MSM of Colour (MOC), 23% African American, 33% Latino, 22% Asian, age range: 19–30 yrs (31, 95%), 31–40 yrs (45%), 4–60 yrs (22, 1%); Sample size: 109	(1) Secondary data analysis of a controlled intervention trial (2) “PCC” (Personalized Cognitive Counselling) intervention + HIV test (3) Standard HIV testing counselling + HIV test	Gold’s Model of “on-line” vs “off-line” self-appraisal of risk behaviour; Model of Relapse Prevention	(1) Health provider (2) Individual (3) 1 session (4) 50 min (5) Training+ regular supervision + review of audiotaped sessions + satisfaction with the intervention questionnaire + multicultural factors addressed	6–12 months	(1) Effectiveness of the intervention on reducing UAI with a non-steady partner or unknown serostatus partner, at 6-months, and to a lesser degree, at 12-months follow-up.
**Eaton et al. 2016** [56]	Community-based research site, USA	MSM; 88.2% African American, 6.2% White, 0.1% Asian, 4.8% other, mean age: 34.5 yrs; Sample size: 600	(1) Randomized controlled trial (2) Intervention (3) Standard HIV testing counselling	Conflict Theory of Decision Making (CTDM); Motivational Interviewing principles	(1) Health provider, video (2) Individual (3) 1 session (4) 45 min (5) Training + audio-recorded and evaluation of the interventions	3, 6, 12 months	(1) Significantly higher proportion of condom-protected sex acts among experimental group’s participants, at follow-ups. (2) No overall effect of the intervention on STI diagnoses.
**Fisher et al. 2006** [24]	2 HIV clinics, USA	PLWH; 45% female (intervention) 39% female (control), 51% African American (intervention) and 25% African American (control); mean age: 43.3 yrs; Sample size: 497	(1) Quasi-experimental study (2) “Options/Opciones” intervention (3) Standard medical care	IMB; Motivational Interviewing principles	(1) Clinicians or nurse + printed materials (2) Individual (3) 2 sessions in average (regular scheduled medical visits) (4) 5–10 min (5) Training + boost training + clinician’s report of the delivery of the steps of the intervention + satisfaction with the intervention questionnaire + multicultural factors and literacy issues addressed	6, 12, 18 months	(1) HIV-infected patients who received the intervention significantly reduced unprotected insertive and receptive vaginal and anal intercourse and insertive oral sex, at 18-months follow-up.
**Gilbert et al. 2008** [42]	5 HIV clinics, USA	PLWH; 79% men, mean, 42% drug users, mean age: 43.9 (intervention group) and 44.3 (control group) yrs; Sample size: 476	(1) Randomized controlled trial (2) “Positive Choices/Video Doctor” intervention (3) Standard medical care	Motivational Interviewing principles	(1) Health provider + computer + video + printed materials (2) Individual (3) 1 session + 1 boost (4) 24 min (5) Cueing sheets used and sign by providers + satisfaction with the intervention questionnaire	3–6 months	(1) Significant reduction of unprotected sex in the intervention group compared with control, at 6- months follow-up.
**Gil-Lario et al. 2014** [18]	University’s lab setting, Spain	Adolescents and young people; 100% Spanish young women, 92% heterosexual, mean age: 21,3 yrs; Sample size: 167	(1) Randomized controlled trial (2) “Informative talk” intervention, or “Attitudinal discussion” intervention, or “Role-play” intervention, or “Fear induction” intervention, or “Informative website” intervention (3) No intervention	IMB	(1) Health provider; health provider + web; health provider + video + printed materials (2) Group (3) 1 session (4) 60 min (5) Cultural factors addressed	4 months	(1) Better results in safe sex behaviour for all female participants. “Attitudinal discussion” has facilitated the best progress of HIV risk factors, at 1- and 4-months follow-ups.
**Grimley et al. 2009** [53]	STI clinic, USA	STI clinic patients; 59, 5% women (intervention group); 54,2% (control group); 90,4% African American (intervention group), 87,7% (control group); mean age: 24,7 yrs (intervention group) and 25,14 yrs (control group); Sample size: 430	(1) Randomized controlled trial (2) Intervention (3) No intervention	Trans theoretical Model (TMM)/Stages of Change	(1) Computer (2) Individual (3) 1 session (4) 15 min (5) Literacy issues addressed	6 months	(1) 32% of intervention group reported consistent condom use, versus 23% in the comparison group, at 6-months follow-up. (2) The combined Neisseria gonorrhoeae and Chlamydia trachomatis incidence declined to 6% in the intervention group versus 13% in the comparison group, at 6-months follow-up.
**Kalichman et al. 2007** [34]	STI clinic, South Africa	STI clinic patients; 100% alcohol users, 85,5% men; mean age: 29,3 yrs; Sample size: 143	(1) Randomized controlled trial (2) HIV/AIDS counselling (3) HIV educational intervention	IMB	(1) Health provider + printed materials (2) Individual (3) 1 session (4) 60 min (5) Training + flipchart to guide the counsellor + weekly supervision + multicultural factors and literacy issues addressed	3, 6 months	(1) Participants who received the experimental intervention demonstrated more than a 25% increase in condom use and a 65% reduction in unprotected intercourse, at 6-months follow-up.
**Kalichman et al. 2011** [35]	STI clinic, South Africa	STI clinic patients; 67% men, 33% women, 93% Black, mean age: 29,2 yrs; Sample size: 617	(1) Random clinical Trial (2) HIV/AIDS counselling (3) HIV educational intervention	IMB	(1) Health provider (2) Individual (3) 1 session (4) 60 min (5) Training + flipchart to guide the counseling + weekly supervision + debriefing meetings + multicultural factors and literacy issues addressed	1, 3, 6, 9, 12 months	(1) Significant reductions in unprotected vaginal and anal intercourse among participants who received the HIV/AIDS counselling, relative to members of the control condition, at 1-, 3- and 6 -months follow-ups. (2) In participants who received the HIV/AIDS counselling there were 24% fewer incident STIs, at 1-, 3- and 6 -months follow-ups.
**Kiene et al. 2006** [43]	University’s lab setting, USA	Adolescents and young people; College students; 70% female; 98% heterosexual; 81% Caucasian; mean age: 18.6 yrs; Sample size: 157	(1) Randomized controlled trial (2) Intervention (3) No intervention	IMB; Motivational Interviewing; Stages of Change	(1) Computer (2) Individual (3) 1 session + 1 boost session (4) 15–40 min + 10–20 min (boost session) (5) Not reported	1 month	(1) Participants who interacted with the computer-delivered HIV/AIDS risk reduction intervention exhibited a significant increase in risk reduction behaviour, at 1-month follow-up.
**Kiene et al. 2016** [32]	General hospital, Uganda	PICT patients; 49.7% women (intervention, 51.2% women (control), mean age: 33 yrs; Sample size: 333	(1) Controlled trial (2) Intervention (3) Standard HIV testing counselling	IMB; Motivational Interviewing principles	(1) Health provider (2) Individual (3) 1 session (4) 10–15 min (5) Training + audio recorded of interventions + health provider self-report	3, 6 months	(1) Intervention showed 1.5–2.4 times greater decreases in high risk sexual behaviour, at 6 months follow-up.
**Latka et al. 2000** [30]	STI clinic, USA	STI clinic patients; 100% women, 90,7% Black (hierarchy message); 96,8% Black (male condom message) and 87,5% Black (female condom message); mean age: 27.8 yrs; Sample size: 292	(1) Unclear (2) “Male condom” intervention, or “Male condom” intervention + “Female condom” intervention, or “Male condom” intervention + “Hierarchical message” intervention	Hierarchical Counselling Model	(1) Health provider + video + printed materials (2) Group or individual (3) 1 session (4) 10–20 min (group delivery) or 20–30 min (individual delivery) (5) Training	2 weeks, 4, 6 months	(1) Hierarchy counselling was associated with a significant increase in condom use with steady partners, at 2 weeks, 4-months and 6- months follow-ups.
**Lightfoot et al. 2010** [25]	6 medical clinics, USA	PLWH; 91% male, 47% White, 20% Black, 33% Hispanic; mean age: 41,8 yrs; Sample size: 529	(1) Quasi-experimental study (2) Computer-delivered intervention, or provider-delivered intervention (3) Standard medical care	Motivational Interviewing principles	(1) Physicians, nurses, case managers, health educators; computer (2) Individual (3) 2,2–2,9 session in average (during regular scheduled medical intervention) (4) 5–15 min (health provider delivery); 10 min (computer delivery) (5) Training + pocked-sized version of the protocol to be used during sessions + quality assurance form completed by patients + boost training	3–30 months	(1) Participants significantly decreased their number of unprotected acts with HIV- /unknown sexual partners in both experimental conditions, when compared with the standard care participants. The computer condition was better at reducing unprotected sexual acts with HIV-/unknown partners, and at reducing the number of unprotected acts overall when compared with the provider-delivered intervention and standard of care condition.
**Newmann et al. 2011** [31]	STD clinic USA, Puerto Rico	STI clinic patients; 51,5% female, 48,5% males, 50,9% Latino, 4,1 Black, 0,8% White; mean age: 29,3 yrs; Sample size: 3365	(1) Controlled trial (2) Intervention (3) Standard medical care and condom distribution	Health Belief Model; Theory of Reasoned Action	(1) Health provider + video + printed materials (2) Group (3) 1 session (4) 40–60 min (5) Training + post session recorded data + “in vivo” observations + multicultural factors and literacy issues addressed	17 months (in average)	(2) Participants enrolled during the intervention were significantly less likely to have an incident STD reported to the surveillance, at an average of 17-months.
**O’Donnell et al. 1998** [54]	STI clinic, USA	STI clinic patients; 100% men, 62% African-American, 37,9% Hispanic; mean age: 30 yrs; Sample size: 2004	(1) Randomized controlled trial (2) Video intervention, or Video + discussion intervention (3) Standard-of-care STD counselling	Health Belief Model; Theory of Reasoned Action	(1) Video; health provider + video (2) Group (3) 1 session (4) 20 min (video), or 45 min (health provider + video) (5) Multicultural factors addressed	17 months (in average)	(2) Rate of new infection was significantly lower among those exposed to video-based prevention education than among controls. No significant differences in rates of infection between those who viewed the video only and those who viewed the video followed by interactive group discussion, at an average of 17- month follow-up.
**O’Donnell et al. 2014** [44]	Community Health Center, USA	MSM; 100% self-identified as Latino men, 64% gay, 30,4% bisexual, mean age: 36,6 yrs; Sample size: 370	(1) Randomized controlled trial (2) “No buscar excusas” intervention + HIV test (3) Non-attention condition +HIV test	Health Belief Model; Theory of Reasoned Action	(1) Health provider + video (2) Group (3) 1 session (4) 45 min (5) Multicultural factors addressed	3 months	(1) Decrease in unprotected intercourse in the intervention group compared to controls, at 3- months follow-up.
**Orr et al. 1996** [55]	Family Planning and STD clinic, USA	Adolescents and young people; 100% women, 55% African American; mean age: 17.8 yrs; Sample size: 209	(1) Randomized controlled trial (2) Intervention (3) Standard STD education	Health Belief Model	(1) Research assistant + printed materials (2) Individual (3) 1 session (4) 10–20 min (5) Not reported	5, 7 months	(1) Participants who received the experimental intervention reported increased use of condoms by their sexual partners for protection against sexually transmitted diseases and for vaginal intercourse. (2) Intervention did not reduce the incident rate of this STD infection.
**Patterson et al. 2008** [38]	Private clinics and community health Centers, Mexico	FSW; 100% female sex workers, 100% Mexican, mean age: 33,5 yrs; Sample size: 924	(1) Randomized controlled trial (2) “Mujer segura” intervention (3) Standard HIV counselling	Social cognitive Theory; Theory of Reasoned Action; Motivational Interviewing principles	(1) Health provider (2) Individual (3) 1 session (4) 35 min (5) Training + multicultural factors addressed	4 months	(1) Concomitant increases in the number and percentage of protected sex acts and decrease in number of unprotected sex acts with clients was reported in the intervention group, at 6- months follow-up. (2) A 40% decline in cumulative transmitted illness incidence in the intervention group was observed, at 6-months follow-up.
**Pitpitain et al. 2014** [29]	STI clinic, South Africa	STI clinic patients; 67% men, 33% women; 93% African American, mean age: 29,2 yrs; Sample size: 617	(1) Secondary analysis of random clinical trial (2) Educational component + motivational component + behavioural skills component + *AUDIT* component (3) HIV educational component	IMB	(1) Health provider (2) Individual (3) 1 session (4) 60 min (5) Weekly supervision + debriefing meetings + flipchart to guide the counsellor + multicultural factors and literacy issues addressed	3, 6, 9, 12 months	(1) The intervention indirectly affected sexual behaviour through alcohol-related constructs, but not IMB constructs, 12 months after the intervention. Alcohol use and related factors play critical roles in explaining HIV and STIs risk reduction intervention effects.
**Richardson et al. 2004** [45]	6 HIV clinics, California, USA	PLWH; 86,2% male, 73,72% MSM, 40,8% White, 37,7% Hispanic, 15,6% African-American, mean age: 38,5 yrs; Sample size: 585	(1) Randomized controlled trial (2) “Partners for Health/ gain frame” intervention, or “Partners for Health/ loss frame “intervention (3) No intervention	Message Framing Theory; Mutual Participation; Trans theoretical Model/Stages of Change	(1) Health provider (2) Individual (3) 1 session (4) 3–5 min (5) Training + boost training + satisfaction with the intervention survey	3 months	(1) Among participants who had two or more sex partners at baseline, UVA (unprotected anal or vaginal intercourse) was reduced 38% among those who received the loss-framed intervention, at 3-months follow-up. No significant changes were seen in the gain-framed arm. No effects were seen in participants with only one partner at baseline.
**Simbayi et al. 2004** [36]	STI clinic, South Africa	STI clinic patients; 65,7% men, 33,7% women; 97% indigenous Africans, mean age: 27.5 yrs; Sample size: 228	(1) Randomized clinical trial (2) HIV risk reduction counselling (3) HIV education intervention	IMB	(1) Health provider (2) Individual (3) 1 session (4) 60 min (5) Training + flipchart used to guide the counsellor session + weekly supervision + multicultural factors and literacy issues addressed	1, 3 months	(1) Significantly greater risk reduction practices, lower rates of unprotected intercourse, and greater likelihood of receiving HIV testing among the counselling participants, at 3-month follow-up.
**Strathdee et al. 2013** [39]	2 NGO offices and mobile units providing HIV and STIs testing, Mexico	FSW; 100% female sex workers who inject drugs, mean age: 34 (Tijuana) and 33 yrs (Ciudad Juarez); Sample size: 584	(1) Randomized controlled trial (2) Didactic injection intervention + didactic sexual risk intervention, or interactive injection risk intervention + didactic sexual risk intervention, or interactive sexual risk reduction intervention (“Mujer mas segura”) + didactic injection risk intervention, or interactive injection risk intervention + interactive sexual risk intervention (“Mujer mas segura”)	Social-Cognitive Theory; Theory of Reasoned Action; Motivational Interviewing principles	(1) Health provider + video + printed materials (2) Individual (3) 1 session (4) 60 min (5) Training + taped sessions with corrective action + boost training + multicultural factors and literacy issues addressed	4, 8, 12 months	(2) In both cities, the intervention significantly reduced HIV/STI incidence, at 12-months follow-up.
**Warner et al. 2008** [47]	3 STI clinics, USA	STI clinic patients; 69% male, 30% female, 1% transgender, 46% White, 25% Hispanic, 18,5% Black, age: 69% ≥ 25 yrs, 31% < 25 yrs; Sample size: 38 635	(1) Multisite controlled trial (2) “Safe in the City” video intervention (3) Educational pamphlets + condoms distribution	IMB; Social Cognitive Theory; Theory of Planned Behaviour	(1) Video + printed materials (2) Individual (in clinics’ waiting rooms) (3) Exposure times not reported (4) 23 min (5) “In vivo” observation + satisfaction with the intervention exit interviews during the formative research + multicultural factors addressed	Up to 24 months (14.8 months in average)	(2) The intervention reduced new infections nearly 10% overall in the three clinics. Patients assigned to the intervention condition had significantly fewer STDs compared to the control condition, during the mean of 14.8 months of observation.
**Wenzel et al. 2015** [27]	3 service sites providing services to homeless women, USA	Homeless women; 56.9% African American, 13.9% Hispanic, 12.6% White, 3.8% Asian, 12.6% others/multi-racial, mean age: 45 yrs; Sample size: 79	(1) Quasi-experimental study (2) Adapted “Sister-to-Sister” intervention (3) HIV information intervention	Social Learning Theory	(1) Health provider + video + printed materials (2) Individual (3) 1 session (4) 40 min (5) Satisfaction with the intervention form + multicultural factors and literacy issues addressed	1 month	(1) Intervention’s participants were significantly more likely to report male or female condom use at last sexual intercourse, at 1-month follow-up.
**Wolfers et al. 2009** [26]	Public Health clinic, Netherlands	MSM; 92% gay, 4,3% bisexual, mean age: 36,6 yrs; Sample size: 281	(1) Quasi-experimental study (2) Intervention (3) No intervention	Theory of Planned Behaviour; Motivational Interviewing principles	(1) Health provider (2) Individual (3) 1 session (4) 15 min (5) Satisfaction with the intervention questionnaire	5–6 months	(1) The intervention had a protective effect on sexual behaviour (UAI) with steady partners, at 5-months follow-up.

AIDS = acquired immunodeficiency syndrome; ART = anti-retroviral therapy; ARV = Anti-retroviral; FSW = female sex worker; HBV = Hepatitis B virus; HIV = human immunodeficiency virus; IMB = Information Motivation Behavioural skills (IMB) Model; MI = Motivational Interviewing; MOC = MSM of colour; MSM = men who have sex with men; NGO = non-governmental organisations; PCC = Personalized Cognitive Counselling; PLWH = people living with HIV; RCT = randomized controlled trial; STI = sexually transmitted infection; STD = sexually transmitted disease; UAI = unprotected anal intercourse, YBMSM = Young Black MSM; PICT = provider-initiated HIV testing and counselling

#### Participants/Population

In most of the studies, participants were STI clinic patients (n = 16), young people or adolescents (n = 5) [18, 37, 43, 55, 59], people living with HIV (PLWH) (n = 5) [24, 25, 33, 42, 45], men who have sex with men (MSM) (n = 7) [26, 28, 41, 44, 46, 49, 56], female sex workers (FSW) (n = 2) [38, 39], PICT (provider-initiated HIV testing and counselling) patients n = 1 [32], and homeless women n = 1 [27]. Sample size ranged from 79 [27] to 38,635 participants [47].

#### Effectiveness and follow-ups

Twenty-six studies compared a behaviour change intervention to prevent HIV, STI and/or unintended pregnancies to a control group receiving no intervention or receiving “standard of care” or a “standard counselling”. In 11 studies, authors compared the effectiveness of specific techniques and/or modes of delivery [18, 25, 29, 30, 37, 39, 45, 46, 48, 51, 54]. Diverse outcome measures were used to assess the behavioural change. The outcome measures were biomedical (i.e. STI reported diagnoses, reduction in the rate of pregnancies, HIV/STI incidence, STI reinfection), behavioural (i.e. number of unprotected vaginal and anal sex acts, percentage of safe sex behaviour, unprotected anal intercourse with steady and with non-steady partners) [25, 26], or a combination of both [27, 35, 38, 48, 49, 52, 53, 55–59]. With a 6 months median, follow-ups ranged from 1 month [27, 43] to 30 months [25]. Nine studies reported a 12-months follow-up, 12 studies reported a 6-months follow-up, three indicated a 9-months follow-up [50, 51, 59], one had a 7 months-follow-up [55], two had a 4-months follow-up [18, 37], three studies indicated a 3-months follow-up [36, 44, 45] and two studies indicated a 1-month follow-up [27, 43]. In five studies, follow-ups reported by authors were up to 17 [31, 54], 18 [24], 24 [47], and 30 months [25].

#### Theoretical background

Following Michie et al., a theoretical basis should represent an integrated summary of the hypothetical causal processes involved in behaviour change [12, 13, 60]. In 34 studies, interventions were theory-based, as they used explicit causal pathways. The most commonly mentioned theoretical framework was the *Information Motivation Behavioural Skills Model of Behaviour Change (IMB)* (n = 12) [18, 24, 29, 33, 34–37, 40, 43, 47, 48, 52, 61, 62]. *Motivational Interviewing (MI)* principles [63] were reported as part of theoretical basis in seven studies [24–26, 32, 33, 38, 42, 43, 56], but these principles were also used in five other studies without being defined as such [29, 34–36, 39]. One study referred to the *Hierarchical Counseling Model* [30], to the *Health Belief Model* [55], to *Social Learning Theory* [27], and one to *Transtheoretical Model /Stages of Change* [53, 64, 65]. Fourteen studies reported several theoretical frameworks such as *Social Cognitive Theory* and *Theory of Reasoned Action* [39, 59, 66]; *IMB* and *Social Learning Theory* [49, 57], *Message Framing Theory*, *Mutual Participation Theory* and *Transtheoretical Model* [45, 64], *Health Belief Model* and *Theory of Reasoned Action* [31, 44, 66, 67], and *Conflict Theory of Decision Making (CTDM)* and *MI* [56]. Three studies based their intervention on *Gold’s Model of “on-line” vs “off line” self-appraisal* of risk behaviour and *Model of Relapse Prevention* [28, 41, 46] and three studies did not describe the behavioural theories that served as the basis of their interventions [50, 51, 58].

#### Delivery and duration

Delivery modes were diverse. In 18 studies the intervention was delivered by a health provider [24, 26, 28, 29, 32, 33–36, 38, 40, 41, 45, 46, 51, 52, 55, 57], in 15 studies by health provider and multimedia tool (computer, web, video, audio tape) [18, 25, 27, 30, 31, 37, 39, 42, 44, 49, 50, 54, 56, 58, 59] and in four studies, the interventions were multimedia-delivered [43, 47, 48, 53]. They were delivered with or without printed materials (i.e. brochures or posters). In one study, the intervention was delivered by a research assistant [55]. The intervention level was individual in 30 studies. In 7 studies, interventions were delivered in group, and in one study both levels of interventions were reported [30]. Briefer interventions were individually delivered by a health provider or a computer [24, 25, 32, 45]. Regarding the number of sessions, 31 studies tested a single-session intervention, three tested a 1 + 1 boost session [42, 43, 58], and three studies reported the efficacy of the intervention after 2 [24], 2.2 [25] and 2.5 [33] sessions in average, and were delivered during the regular medical appointment. The duration of the experimental conditions (n = 53) varied and ranged from 3 minutes to 1 hour. Their median duration was 47 minutes. Twenty-four experimental conditions had a duration range from 50 to 60 minutes, seven experimental conditions had a duration range from 30 to 49 minutes and 20 had a 3 to 25 minutes duration. In Fisher et al. [24] and Richardson et al. [45], the intervention was delivered by clinicians in respectively 5–10 minutes and 3–5 minutes. In Lightfoot et al. [25], the intervention was delivered in 10 minutes by a computer or in 5–15 minutes by a health provider. In one study that used a waiting room video-delivered intervention, the exposure time was not reported but the duration of the video was 23 minutes [47]. Among the 41 control conditions/ineffective interventions, six had a duration ranged from 45 to 60 minutes, fourteen had a duration ranged from 5 [58] to 35 minutes [38]. The median duration was 30 minutes. In 12 control conditions the duration of the intervention was not reported and in the remaining studies, control conditions were not HIV, STI and unintended pregnancies prevention interventions. Table 2 contains the summary of studies and interventions characteristics.

**Table 2:** Studies and interventions characteristics.

**Studies** n = 37	**Countries**	**Populations**
	U.S.A South Africa Spain Mexico Russia Uganda Netherlands	STI clinic patients MSM Adolescents and young people PLWH FSW Homeless women PICT service patients
**Interventions** n = 85	**Effective**; n = 53	**Control/ ineffective**; n = 32
Delivery methods	Health provider Video Audiotape Printed materials Computer Web	Health provider Video Printed materials
Intervention’s level	Individual n = 37 Group n = 16 Individual or group delivery n = 1	Individual n = 26 Group n = 6 Individual or group delivery n = 2
Intensity	1 session n = 47 1+ 1 boost session n = 6	1 session n = 28 1+ 1 boost session n = 4
Duration	Range 5–60 min Median: 47 min Mean: 40.9 min	Range 5–60 min Median: 30 min Mean: 31.1 min

MSM = men who have sex with men; STI = sexually transmitted infections; PLWH = people living with HIV; FSW = female sex workers; PICT = provider-initiated HIV testing and counselling

#### Intervention fidelity

Among the included studies, 34 provided information regarding fidelity, which includes strategies to assess, monitor and enhance intervention fidelity. These strategies, summarized on S1 Table, consisted on video or audio taping the interventions [28, 31, 39, 41, 46, 57, 58], “in vivo” observations [31, 33, 47], exit interviews, satisfaction with the intervention questionnaires [24–28, 41, 42, 45–48, 59], provider’s self-report (i.e. checklist) [24, 32, 42], table flipchart and pocket size manual [24, 25, 29, 34–36, 45, 58], training [24, 25, 28, 30–32, 34–36, 38–41, 45, 46, 52, 57, 58], boost training [24, 39, 45], debriefing meetings [29, 35] and supervision [28, 29, 34–36, 41, 46]. In 22 studies, information regarding strategies to address multicultural factors and literacy issues were also provided. In Eaton et al. [56], counsellors were trained in client-centred counselling and motivational interviewing. In addition, interventions were audio-recorded and evaluated for fidelity. In Simbayi et al. [36], the intervention was completely manualized, and a table top flipchart was used to guide the counsellors through the session content. In Crosby et al. [48], to reliably deliver the intervention, 2-day training session for health educators was provided and through the study period, every 10^th^ intervention was tape-recorded for quality assurance monitoring. To assess the fidelity to the control condition protocol in a General Hospital in Uganda, counselling sessions in the control group were audio recoded, transcribed and translated into English and then coded to determine how many of the protocol steps were completed [32]. Among our included studies, satisfaction with the intervention questionnaires, training, video or audio taping the interventions, supervision and addressing multicultural factors were the most commonly used strategies to assess, monitor and enhance the intervention fidelity.

#### Target behaviour of BCTs

The relevant target behaviour of techniques identified using the BCTTv1 taxonomy was condom use. Depending on the studies, the effect of these techniques on behaviour was measured in terms of decreased unprotected intercourse [24, 33, 34], condom use during the last sex act [37, 27], reduction of proportions of participants reporting unprotected anal intercourses (UAI) with non-steady partners [41], reduction of episodes of unprotected anal or vaginal intercourse [25, 42, 46, 49, 59], consistent condom use [53, 59] or mean number of UAI [46].

### Risk of bias

The risk of bias varied within included studies. The main domain on which, the studies were at high risk of bias was allocation concealment (11 studies scored “high”).

The majority of the studies were low at risk on selective reporting (27 studies scored “low”), and on incomplete outcome data (27 studies scored “low”). More than half of the studies were low at risk on random sequence (19 studies scored “low”) and on blinding of outcomes (19 studies scored “low”). For example, on random sequence, in Crosby et al. [49], participants were randomized (using a computer-generated algorithm) to either the experimental or a standard-of care control condition. Gilbert et al. [42], reported that a high retention was achieved for follow-ups, as 82% of the intervention group and 83% of the control group completed the 6-month follow-up session. Three studies were low at risk on blinding of participants, and at high risk on blinding of personnel, as for example in Kiene et al. [32], who stated that participants, but not study staff, were blinded to the study condition assignment; however, authors did not assess if unbinding occurred.

In 34, 18 and 17 studies, the risk of bias was unclear, respectively on other potential bias, on blinding of participants and personnel, and on blinding outcomes. S2 Table provides the risks of bias of each study.

### Identification of BCTs in brief interventions to prevent HIV, STI and unintended pregnancies

Within the included studies (n = 37) we coded in total 85 interventions according to the BCTTV1 coding guidelines (53 effective interventions and 32 control/ ineffective interventions). Nine control conditions were “no intervention” conditions or were control conditions not encompassing any BCTs related to HIV, STI and unintended pregnancies prevention. S3 Table shows BCTs identified, their frequency of use and delivery methods. Concerning the interrater reliability on agreeing on the presence or the absence of BCTs, Kappa value indicated a high substantial agreement (Cohen’s Kappa = .781) between the coders on their independent set of coding (before the consensual phase). This is interpreted as a low coding bias and a good likelihood of replicability of the findings.

#### Number of BCTs identified

Overall, in effective and control/ineffective interventions, 50 out of 93 BCTs were identified. These 50 BCTs were identified in 15 of the 16 possible clusters of BCTs. In effective interventions, 48 BCTs were identified. The median number of BCTs identified per effective intervention was 9. In control/ineffective interventions, 29 BCTs were identified, and the median number of BCTs per intervention was 6.

#### BCTs frequently identified in effective interventions

Table 3 synthesizes the frequently used BCTs. In effective interventions, 8 BCTs were identified in at least 50% of the interventions. These BCTs were: *1*.*2 “Problem solving (57%) 2*.*2 “Feedback on behaviour”* (55%), *3*.*1 “Social support (unspecified)”* (87%), *4*.*1 “Instructions on how to perform the behaviour”* (87%), *5*.*1 “Information about health consequences”* (81%), *5*.*3 “Information about social and environmental consequences”* (70%), *6*.*1 “Demonstration of behaviour”* (55%) and *9*.*1 “Credible source”* (92%). Each of these frequently used techniques encompassed several delivery methods. For example, 1.*2 “Problem solving”* was delivered by health provider and by computer. In the case of *4*.*1 “Instructions on how to perform the behaviour”*, the delivery methods were: health provider, video, computer, printed materials and web, and regarding the BCT *5*.*1 “Information about health consequences”* the delivery methods were, health provider, video, computer, audiotape, printed materials and web (S3 Table).

**Table 3:** Synthesis of BCTs frequently used in effective and control/ineffective interventions.

BCTs identified n = 50	Effective interventions	Control/ineffective intervention
Number of BCTs identified	48	29
Median number of BCTs per intervention	9	6
Range	3–25	2–12
BCTs frequently* used (%)	1.2 Problem-solving (57%) 2.2 Feedback on the behaviour (55%) 3.1 Social support (unspecified) (87%) 4.1 Instructions on how to perform the behaviour (87%) 5.1 Information about health consequences (81%) 5.3 Information about social and environmental consequences (70%) 6.1 Demonstration of the behaviour (55%) 9.1 Credible source (92%)	3.1 Social support (unspecified) (91%) 4.1 Instructions on how to perform the behaviour (69%) 5.1 Information about health consequences (81%) 5.3 Information about social and environmental consequences (53%) 9.1 Credible source (94%)

BCTs = Behaviour Change Techniques;

*identified in at least 50% of the interventions

#### BCTs frequently identified in control/ineffective interventions

In control/ineffective interventions, 5 BCTs were frequently used: *3*.*1 “Social support (unspecified)”* identified in 91% of control/ineffective interventions, *4*.*1 “Instructions on how to perform the behaviour”* (69%), *5*.*1 “Information about health consequences”* (81%), *5*.*3 “Information about social and environmental consequences”* (53%) and *9*.*1“Credible source” (94%)*. As indicated on Table 3, these 5 BCTs identified as frequently used in control/ineffective interventions are shared with effective interventions.

Regarding delivery formats, S3 Table shows that four of these frequently used BCTs were exclusively delivered by a health provider (*3*.*1 “Social support (unspecified)”* and *9*.*1 “Credible source*”), or health provider and printed materials (*4*.*1 “Instructions on how to perform the behaviour”*, *5*.*1 “Information about health consequences” and 5*.*3 “Information about social and environmental consequences”*), without any multimedia tool.

#### BCTs frequently identified in effective and control/ineffective interventions according to the intensity, the duration, the delivery and the level of the intervention

Further to the examination of the frequently used BCTs in effective and control/ineffective interventions to prevent HIV, STI and unintended pregnancies, four factors that influence interventions’ outcomes were considered: the intensity, the duration, the delivery and the level of the intervention. Hence, the frequency of use of each BCTs was descriptively assessed in (1) interventions delivered in 1 session vs interventions delivered in 1 + 1 boost session; (2) interventions with a duration up to 40 min (up to 31 min for control/ineffective interventions) vs longer interventions, (3) interventions delivered by a health provider (with or without printed materials) vs interventions delivered by a health provider in conjunction with multimedia tools (computer, video, web, audiotape); and (4) individual vs group-delivered interventions. In Tables 4 and 5 and in S4 Table are summarized the findings of these additional assessments, made within effective interventions and within control/ineffective interventions.

**Table 4:** Frequent BCTs identified in effective interventions according to the intensity, duration, delivery and level of the intervention.

Effective interventions; N = 53	Intensity		Duration		Delivery		Level of the intervention	
	1 session; n = 46	1 + 1 boost session; n = 7	> 40 min; n = 30	≤ 40 min; n = 23	Health provider-delivered; n = 30	Health provider and multimedia-delivered; n = 23	Individual level; n = 37	Group level; n = 16
Frequent BCTs*								
1.1 Goal setting (behaviour)		x						
1.2 Problem solving	x	x	x		x	x	x	
1.4 Action planning	x							
1.8 Behavioural contract	x							
2.2 Feedback on the behaviour	x	x	x	x	x		x	
3.1 Social support (unspecified)	x	x	x	x	x	x	x	x
4.1 Instructions on how to perform the behaviour	x	x	x	x	x	x	x	x
5.1 Information about health consequences	x	x	x	x	x	x	x	x
5.3 Information about social and environmental consequences	x	x	x	x	x	x	x	x
6.1 Demonstration of the behaviour	x	x	x	x		x	x	x
9.1 Credible source	x	x	x	x	x	x	x	x

BCTs = Behaviour Change Techniques;

*identified in at least 50% of the interventions

**Table 5:** Frequent BCTs identified in control/ineffective interventions according to the intensity, duration, delivery and level of the intervention.

Control/ineffective interventions; N = 32	Intensity		Duration		Delivery		Level of the intervention	
	1 session n = 28	1 + 1 boost session; n = 4	≤ 31min; n = 28	> 31 min; n = 4	Health provider-delivered; n = 26	Health provider and multimedia-delivered n = 5	Individual level; n = 26	Group level; n = 6
**Frequent BCTs**								
1.1 Goal setting (behaviour)								
1.2 Problem solving				x				
1.3 Goal setting (outcome)				x				
1.4 Action planning								
1.8 Behavioural contract								
2.2 Feedback on the behaviour				x				
3.1 Social support (unspecified)	x	x	x	x	x	x	x	x
4.1 Instructions on how to perform the behaviour	x		x	x	x	x	x	
5.1 Information about health consequences	x		x	x	x	x	x	
5.2 Salience of consequences						x		x
5.3 Information about social and environmental consequences	x		x	x		x	x	x
6.1 Demonstration of the behaviour				x		x		
9.1 Credible source	x	x	x	x	x	x	x	x
10.4 Social reward				x				
12.5 Adding objects to the environment								x

BCTs = Behaviour Change Techniques;

*identified in at least 50% of the interventions

The examination of effective interventions, according to their duration showed that in briefer effective interventions (which duration was 40 min or less), the BCT *1*.*2 “Problem solving*” was not frequently used.

Findings also showed that in group-delivered effective interventions, the BCTs *1*.*2 “Problem solving”* and *2*.*2 “Feedback on the behaviour”* weren’t frequently used. Thus, these two techniques addressing personal concerns and providing a tailored feedback regarding the behaviour were only frequently used in effective interventions individually delivered.

Findings also indicated that when the effective interventions were delivered by a health provider (without any multimedia tool), the BCT *6*.*1 “Demonstration of the behaviour”* wasn’t frequently used.

With respect to control/ineffective interventions, Table 5 shows that in control/ineffective interventions that were group-delivered, the BCT frequently used to provide information was *5*.*3 “Information about social and environmental consequences”*. Another notable finding was that, in longer control/ineffective interventions (delivered in more than 31 min), 10 BCTs were frequently used. Among these BCTs were *1*.*2 “Problem solving”*, *2*.*2 Feedback on the behaviour” and 6*.*1 “Demonstration of the behaviour”*, despite the fact that the interventions were control/ineffective. Findings also showed that only in interventions delivered in group and in interventions delivered by a health provider in conjunction with a multimedia tool, the BCTs *5*.*2 “Salience of consequences”* was frequently used. Lastly, findings showed that condom distribution was frequently provided in group-delivered control/ineffective interventions (*12*.*5 “Adding objects to the environment”*).

## Discussion

### Technical heterogeneity and complexity of effective interventions

Findings on the number of BCTs (48) identified in effective interventions showed that a large scope of different BCTs can be used to target one single behaviour. This indicates that the techniques used in these interventions were relatively heterogeneous and that interventions with different technical curricula can be effective. The number of different theoretical bases in which interventions are grounded (13 theoretical frameworks) could be an explanation for the large scope of BCTs identified in effective interventions. Another explanation is the possibility that different techniques based on different theoretical constructs achieved similar outcomes through different mechanisms of action [68]. The large scope of BCTs used in effective interventions can also be explained by the variety of key populations targeted by effective interventions. Further research should clarify to what extent the variation of populations targeted by these interventions is responsible for the broad array of BCTs identified in effective interventions.

With a respective range from 3 to 25 BCTs per effective intervention, results also indicated that these interventions were variable in terms of technical complexity, and highlight that an intervention encompassing only a few techniques can be effective [53]. However, as indicated in some interventions’ manuals, despite the fact that health providers were trained to deliver a very large scope of techniques [24, 33], it is plausible that all these techniques may have not been used systematically, since interventions were often designed to be adjusted to the clients’ needs and concerns.

### BCTs frequently used in effective interventions

In effective interventions, 8 BCTs were frequently identified. This “set” of BCTs encompasses 5 shared techniques (*3*.*1 “Social support”*, *4*.*1 “Instructions on how to perform the behaviour”*, *5*.*1 “Information about health consequences”*, *5*.*3 “Information about social and environmental consequences”* and *9*.*1 “Credible source*”) that were not unique to effective interventions, and 3 frequently used BCTs that were unique to effective interventions (*1*.*2 “Problem solving”*, *2*.*2 “Feedback on behaviour*” and *6*.*1 “Demonstration of the behaviour*”). It is plausible that these 3 unique BCTs are potentially critical to the technical content embedded in effective interventions. Our results are consistent with previous findings supporting that effective interventions increasing condom use comprise educational information, attitudinal arguments to promote condom use (i.e. discussions of the positive implication of using condoms for the health of the partner/s or for romantic relationships), arguments designed to model behavioural skills (i.e. what to do when partners do not want to use a condom, when recipients or their partner/s are sexually excited and when alcohol or drugs are involved) and condom use modelled behavioural skills [6, 20, 69, 70].

With respect to the 5 frequent BCTs that were not unique to the effective interventions, these techniques were the only BCTs identified in two effective interventions of our sample (“Informative talk” and “Informative website group”) [18] (without any additional technique). Consequently, we cannot conclude that these techniques are ineffective techniques and/or not necessary to an intervention to achieve behaviour change. Consistent with previous findings on interventions content that most successfully increased condom use [69, 70] (i.e. providing educational information and arguments to promote positive attitudes towards condom use), we can hypothesize that the 5 techniques not unique to effective interventions, contribute to the intervention’s effectiveness when used with BCTs that have been identified as frequently used in interventions that achieve significant behaviour change.

Regarding effective interventions group-delivered, and the fact that in this small sample of effective interventions (n = 16), the BCTs *1*.*2 “Problem solving”* and *2*.*2 “Feedback on the behaviour”* weren’t frequently used (without impeding the effectiveness of these interventions), it is plausible, that personal concerns may have been addressed by using other techniques that have impacted the mechanism of behaviour change and thus achieved positive outcomes.

This brings us to hypothesise that the BCTs *1*.*2 “Problem solving”*, *2*.*2 “Feedback on behaviour*” and *6*.*1 “Demonstration of the behaviour*” plausibly may be working additively or in conjunction with the 5 shared BCTs frequently identified across all interventions, and that effective interventions should consist of a “core set” of 8 BCTs: (a) providing or arranging social support that can include some counselling and encouragement to support the desired behaviour *(3*.*1 “Social support (unspecified)*”), (b) informing and instructing about target behaviour *(5*.*1 “Information about health consequences”*, *5*.*3 “Information about social and environmental consequences*” and *4*.*1 “Instructions on how to perform the behaviour”*), and (c) addressing the individual’s concerns in a tailored way, by providing informative or evaluative feedback on the individual’s performance of the target behaviour *(2*.*2 “Feedback on behaviour”*), and by analysing or prompt the person to analyse factors influencing their personal behaviour, and subsequently, generate tailored strategies with the participant, and provide observable samples of the behaviour, in order to overcome barriers to safer behaviours and/or increase desired behaviour facilitators (*1*.*2* “*Problem solving*” and *6*.*1 “Demonstration of behaviour”*). Lastly, (d) effective interventions were usually delivered by a source generally agreed as credible (*9*.*1* “*Credible source”*), such as a health provider (in person or/and in conjunction with a multimedia tool). Relevant to this point, the effectiveness of these techniques is particularly related to the provider’s communicational skills necessary to establish a non-threatening context, to discuss sexuality concerns, to offer an empathic understanding as well as an unconditional regard supporting the client’s behaviour change.

### Limitations and further research

The exclusion of brief and effective interventions delivered in more than one session or interventions which duration exceeded 60 min, leads to a small number of studies and interventions. It is also possible that effective BCTs are different for multi-session and longer interventions.

Only 17 studies of our sample reported biomedical outcomes. One important limitation of our review is that the behavioural outcomes were based on self-reported data. These outcomes may be especially vulnerable to biases related to social desirability, given to personal nature of sexual behaviour and there is clearly a need for the greater inclusion of biomedical outcomes in efficacy trials of brief interventions.

Our results are based on a sample of studies in which populations as sex workers, people living with HIV, MSM, PICT patients, homeless women, adolescents and young people, were less represented than STI clinic patients. It can be a source of population selection bias and therefore limits further extrapolation of the findings.

Our review is based on BCTs identification following the BCTTv1 taxonomy and guidelines, and thus constrained by the clarity and the completeness of the interventions’ descriptions. A poorly or well-reported intervention might respectively lead to under or overrepresentation of some BCTs. This material-related bias was balanced by consulting supplementary materials, when available. However, in this review, BCTs were mostly coded from published and available information rather than from intervention protocols.

Regarding the interventions delivered using multimedia, BCTs identification was particularly difficult and challenging as videos were not always accessible. This was a limitation to identify all the BCTs that were actually delivered during the interventions.

Owing to the heterogeneity of interventions’ content, design and type of outcome measures, we didn’t combine the statistical technique of meta-regression with the BCTTv1 analysis. Therefore, the conclusions of our work must be considered as preliminary indications on potentially effective techniques to achieve behaviour change, as it is not possible to discern the effect of each BCT on the effectiveness of the interventions, or to examine the cumulative or additive effect of BCTs.

Most of the included studies provided information on intervention fidelity. Nevertheless, this information was not equally detailed across studies and not all the studies provided information regarding intervention’s fidelity of control conditions. It is plausible that differences in fidelity exist between experimental and control conditions. This may have led to the ineffectiveness of controls. This limitation of highlights the need for caution when interpreting the results.

This systematic review did not investigate other important factors influencing the effectiveness of a unique BCT or combination of BCTs, effectiveness of BCTs according to the target populations, acceptability and feasibility of a BCT in different socio-cultural contexts or settings, neither factors such as cost-effectiveness of the interventions. Each of these aspects requires further research.

## Conclusion

In this review, we identified and synthesized technical content of brief interventions effective to prevent HIV, STI and unintended pregnancies in a standardized and replicable way.

Our findings suggest that a frequently identified set of BCTs, deliverable in a variety of formats, is potentially associated with effectiveness of HIV, STI and unintended pregnancy prevention–allowing individuals to achieve their sexual health goals. Hypothetically, an effective sexual health intervention, aiming at addressing issues related to main risks that arise from unsafe sexual practices should comprise, at least, this set of BCTs. The use of these techniques may be particularly beneficial for interventions delivered in settings with time-constraints and limited resources.

It is timely and important to develop and test targeted brief behaviour interventions as a means to strengthen health care providers’ communication skills, and thereby improving the quality of sexual health services [1].

This study in conjunction with other systematic reviews [6, 7, 69, 71–81] support the needs for further studies to assess effectiveness, acceptability, feasibility and cost-effectiveness of BCTs that have to be tailored to the needs of different populations in their specific contexts.

## Supporting information

S1 Table Intervention fidelity. (DOCX)

S2 Table Risk of bias. (DOCX)

S3 Table BCTs identified and modes of delivery. (DOCX)

S4 Table Summary of BCTs frequently used in interventions according to duration, intensity, delivery and level of the intervention. (DOCX)

S1 File Search strategy. (DOCX)

S2 File List of 93 BCTs and clusters. (DOCX)

S3 File Prima checklist. (DOC)
