# Supplementary material for: Behaviour change techniques in brief interventions to prevent HIV, STI and unintended pregnancies: A systematic review
Source: PLoS One. 2018 Sep 27;13(9):e0204088. doi: 10.1371/journal.pone.0204088 (PMC6159869; doi:10.1371/journal.pone.0204088)
Supplement: S1 Table — (DOCX) [file pone.0204088.s001.docx]

**S 1 Table. Intervention fidelity.**

| Studies | Strategies to assess, monitor and enhance treatment fidelity | | | | | | | | | | |
| --- | --- | --- | --- | --- | --- | --- | --- | --- | --- | --- | --- |
|  | Videotaping, audiotaping of the intervention | In vivo observation | Participant self-report | Provider self-report (checklist) | User-friendly scripted curriculum or manual (i.e. table flipchart) | Training | Boost training, | Debriefing meetings | Supervision | Multicultural factors considered in the development and/or delivery of the intervention | Literacy |
| Abdala et al. ^[40]^ |  |  |  |  | x | x |  |  |  | x | x |
| Artz et al. ^[58]^ | x |  |  |  | x | x |  |  |  |  |  |
| Ballester-Arnal et al. ^[37]^ |  |  |  |  |  |  |  |  |  | x |  |
| Boekeloo et al. ^[59]^ |  |  | x |  |  | x |  |  |  |  |  |
| Carey et al. ^[48]^ |  |  | x |  |  |  |  |  |  | x |  |
| Cohen et al. ^[50]^ |  |  |  |  |  |  |  |  |  | x |  |
| Cohen et al. ^[51]^ |  |  |  |  |  |  |  |  |  |  |  |
| Cornman et al. ^[33]^ |  | x |  |  |  | x |  |  | x | x | x |
| Crosby et al^. [52]^ |  |  |  |  |  | x |  |  |  | x |  |
| Crosby et al. ^[57]^ | x |  |  |  |  | x |  |  | x | x |  |
| Crosby et al. ^[49]^ |  |  |  |  |  |  |  |  |  | x | x |
| Dilley et al. ^[46]^ | x |  | x |  |  | x |  |  | x |  |  |
| Dilley et al^. [41]^ | x |  | x |  |  | x |  |  | x |  |  |
| Dilley et al. ^[28]^ | x |  | x |  |  | x |  |  | x | x |  |
| Eaton et al. ^[56]^ | x |  |  |  |  | x |  |  |  |  |  |
| Fisher et al. ^[24]^ |  |  | x | x | x | x | x |  |  | x | x |
| Gilbert et al. ^[42]^ |  |  | x | x |  |  |  |  |  |  |  |
| Gil-Lario et al. ^[18]^ |  |  |  |  |  |  |  |  |  | x |  |
| Grimley et al. ^[53]^ |  |  |  |  |  |  |  |  |  |  | x |
| Kalichman et al. ^[34]^ |  |  |  |  | x | x |  |  | x | x | x |
| Kalichman et al. ^[35]^ |  |  |  |  | x | x |  | x | x | x | x |
| Kiene et al. ^[43]^ |  |  |  |  |  |  |  |  |  |  |  |
| Kiene et al. ^[32]^ |  |  |  | x |  | x |  |  |  |  |  |
| Latka et al. ^[30]^ |  |  |  |  |  | x |  |  |  |  |  |
| Lightfoot et al. ^[25]^ |  |  | x |  | x | x | x |  |  |  |  |
| Newmann et al. ^[31]^ | x | x |  |  |  | x |  |  |  | x | x |
| O’Donnell et al. ^[54]^ |  |  |  |  |  |  |  |  |  | x |  |
| O’Donnell et al. ^[44]^ |  |  |  |  |  |  |  |  |  | x |  |
| Orr et al. ^[55]^ |  |  |  |  |  |  |  |  |  |  |  |
| Patterson et al. ^[38]^ |  |  |  |  |  | x |  |  |  | x | x |
| Pitpitain et al. ^[29]^ |  |  |  |  | x | x |  | x | x | x | x |
| Richardson et al. ^[45]^ |  |  | x |  |  | x | x |  |  |  |  |
| Simbayi et al. ^[36]^ |  |  |  |  | x | x |  |  | X | x | x |
| Strathdee et al. ^[39]^ | x |  |  |  |  | x | x |  |  | x | x |
| Warner et al. ^[47]^ |  | x | x |  |  |  |  |  |  | x |  |
| Wenzel et al. ^[27]^ |  |  | x |  |  |  |  |  |  | x | x |
| Wolfers et al. ^[26]^ |  |  | x |  |  |  |  |  |  |  |  |
| Total studies using strategies | 8 | 3 | 12 | 3 | 8 | 22 | 4 | 2 | 9 | 22 | 13 |
