# Supplementary material for: Behaviour change techniques in brief interventions to prevent HIV, STI and unintended pregnancies: A systematic review
Source: PLoS One. 2018 Sep 27;13(9):e0204088. doi: 10.1371/journal.pone.0204088 (PMC6159869; doi:10.1371/journal.pone.0204088)
Supplement: S2 Table — (DOCX) [file pone.0204088.s002.docx]

**S2 Table. Risk of bias.**

| Reference | Risk of bias |
| --- | --- |
| Abdala et al. 2013 ^[40]^ | - RS: unclear - AC: unclear - BP: low - BO: unclear - IO: low - SR: low - OR: unclear |
| Artz et al. 2005 ^[58]^ | - RS: low - AC: low - BP: unclear - BO: low - IO: low - SR: low - OR: unclear |
| Ballester-Arnal et al. 2014 ^[37]^ | - RS: low - AC: low - BP: unclear - BO: low - IO: low - SR: low - OR: unclear |
| Boekeloo et al. 1999 ^[59]^ | - RS: unclear - AC: unclear - BP: unclear - BO: low - IO: low - SR: low - OR: unclear |
| Carey et al. 2014 ^[48]^ | - RS: unclear - AC: unclear - BP: unclear - BO: unclear - IO: unclear - SR: unclear - OR: unclear |
| Cohen et al. 1992 ^[50]^ | - RS: high - AC: high - BP: unclear - BO: low - IO: low - SR: low - OR: unclear |
| Cohen et al. 1992 ^[51]^ | - RS: low - AC: low - BP: high - BO: low - IO: unclear - SR: low - OR: unclear |
| Cornman et al. 2008 ^[33]^ | - RS: unclear - AC: unclear - BP: unclear - BO: unclear - IO: low - SR: low - OR: unclear |
| Crosby et al. 2009 ^[52]^ | - RS: low - AC: low - BP: low - BO: low - IO: low - SR: low - OR: unclear |
| Crosby et al. 2014 ^[57]^ | - RS: low - AC: low - BP: Participant =low; Personnel= low - BO: unclear - IO: low - SR: low - OR: unclear |
| Crosby et al. 2017 ^[49]^ | - RS: low - AC: low - BP: Participant= low, Personnel= low - BO: unclear - IO: low, low retention rate but differential attrition not observed - SR: low - OR: unclear |
| Dilley et al. 2002 ^[46]^ | - RS: low - AC: low - BP: high - BO: unclear - IO: low - SR: unclear - OR: unclear |
| Dilley et al. 2007 ^[41]^ | - RS: low - AC: low - BP: low - BO: low - IO: low - SR: low - OR: unclear |
| Dilley et al. 2010 ^[28]^ | - RS: low - AC: low - BP: low - BO: low - IO: low - SR: low - OR: unclear |
| Eaton et al. 2016 ^[56]^ | - RS: low - AC: low - BP: personnel= unclear; Participants= unclear - BO: unclear - IO: low, outcomes reported - SR: low - OR: unclear |
| Fisher et al. 2006 ^[24]^ | - RS: high - AC: high - BP: high - BO: unclear - IO: low - SR: high - OR: high |
| Gilbert et al. 2008 ^[42]^ | - RS: low - AC: low - BP: low - BO: low - IO: low - SR: low - OR: unclear |
| Gil-Lario et al. 2014 ^[18]^ | - RS: unclear - AC: unclear - BP: unclear - BO: unclear - IO: unclear - SR: low - OR: unclear |
| Grimley et al. 2009 ^[53]^ | - RS: low - AC: low - BP: low - BO: low - IO: low - SR: low - OR: unclear |
| Kalichman et al. 2007 ^[34]^ | - RS: low - AC: unclear - BP: unclear - BO: unclear - IO: low - SR: unclear - OR: unclear |
| Kalichman et al. 2011 ^[35]^ | - RS: low - AC: unclear - BP: low - BO: low - IO: unclear - SR: unclear - OR: high |
| Kiene et al. 2006 ^[43]^ | - RS: unclear - AC: low - BP: low - BO: low - IO: high - SR: low - OR: unclear |
| Kiene et al. 2016 ^[32]^ | - RS: high - AC: high - BP: Personnel =high; Participants= low-unclear - BO: unclear - IO: high - SR: low - OR: unclear |
| Latka et al. 2000 ^[30]^ | - RS: high - AC: high - BP: Personnel= high; Participants= low - BO: low - IO: high - SR: low - OR: unclear |
| Lightfoot et al. 2010 ^[25]^ | - RS: high - AC: high - BP: Personnel= high; Participants= low - BO: low - IO: low - SR: low - OR: unclear |
| Newmann et al. 2011 ^[31]^ | - RS: high - AC: high - BP: low - BO: low - IO: unclear - SR: low - OR: unclear |
| O’Donnell et al. 1997 ^[54]^ | - RS: low - AC: low - BP: low - BO: low - IO: unclear - SR: low - OR: unclear |
| O’Donnell et al. 2014 ^[44]^ | - RS: unclear - AC: unclear - BP: unclear - BO: low - IO: low - SR: low - OR: unclear |
| Orr et al. 1996 ^[55]^ | - RS: high - AC: high - BP: low - BO: unclear - IO: low - SR: low - OR: unclear |
| Patterson et al. 2008 ^[38]^ | - RS: low - AC: low - BP: unclear - BO: unclear - IO: unclear - SR: unclear - OR: low |
| Pitpitain et al. 2014 ^[29]^ | - RS: low - AC: low - BP: low - BO: high - IO: low - SR: low - OR: unclear |
| Richardson et al. 2004 ^[45]^ | - RS: high - AC: high - BP: Personnel= high; Participants= low - BO: unclear - IO: low - SR: unclear - OR: unclear |
| Simbayi et al. 2004 ^[36]^ | - RS: low - AC: high - BP: unclear - BO: unclear - IO: low - SR: unclear - OR: unclear |
| Strathdee et al. 2013 ^[39]^ | - RS: low - AC: low - BP: unclear - BO: unclear - IO: low - SR: unclear - OR: unclear |
| Warner et al. 2008 ^[47]^ | - RS: high - AC: high - BP: unclear - BO: low - IO: low - SR: low - OR: unclear |
| Wenzel et al. 2015 ^[27]^ | - RS: high - AC: high - BP: unclear - BO: unclear - IO: low - SR: unclear - OR: unclear |
| Wolfers et al. 2009 ^[26]^ | - RS: unclear - AC: unclear - BP: unclear - BO: low - IO: low - SR: low - OR: unclear |

RS: Random Sequence; AC: Allocation; BP: Blinding of participants and personnel; BO: Blinding of outcome; IO: Incomplete outcome data; SR: Selective Reporting; OR: Other potential sources of bias
