# Supplementary material for: Behaviour change techniques in brief interventions to prevent HIV, STI and unintended pregnancies: A systematic review
Source: PLoS One. 2018 Sep 27;13(9):e0204088. doi: 10.1371/journal.pone.0204088 (PMC6159869; doi:10.1371/journal.pone.0204088)
Supplement: S3 Table — (DOCX) [file pone.0204088.s003.docx]

**S3 Table. BCTs identified and modes of delivery.**

| BCTs | Number of effective interventions in which the BCT was identified | % of use in effective interventions  n=53 | Delivery methods | Number of control/ ineffective interventions in which the BCT was identified n=32 | % of use in control/ineffective interventions | Delivery methods |
| --- | --- | --- | --- | --- | --- | --- |
| 1. **Goals and planning** | | | | | | |
| **1.1 Goal-setting (behaviour)** | 13 | 25% | health provider, computer | 2 | 6% | health provider |
| **1.2 Problem-solving** | 30 | 57% | health provider, video, computer | 5 | 16% | health provider |
| **1.3 Goal-setting (outcome)** | 12 | 23% | health provider | 5 | 16% | health provider |
| **1.4 Action planning** | 14 | 26% | health provider, computer | 1 | 3% | health provider |
| **1.5 Review behaviour goal(s)** | 3 | 6% | health provider | 1 | 3% | health provider |
| **1.6 Discrepancy between current behaviour and goal** | 4 | 8% | health provider | 0 | 0% | - |
| **1.7 Review outcome goal(s)** | 2 | 4% | health provider, computer | 0 | 0% | - |
| **1.8 Behavioural contract** | 7 | 13% | health provider, computer | 1 | 3% | health provider |
| **1.9 Commitment** | 5 | 9% | health provider | 0 | 0% | - |
| 1. **Feedback and monitoring** | | | | | | |
| **2.1 Monitoring of behaviour by others without feedback** | 0 | 0% |  | 1 | 3% | health provider |
| **2.2 Feedback on behaviour** | 29 | 55% | health provider, computer | 9 | 28% | health provider |
| **2.3 Self-monitoring of behaviour** | 8 | 15% | computer, printed materials | 1 | 3% | printed material |
| **2.4 Self-monitoring of outcome(s) of behaviour** | 6 | 11% | computer, printed materials | 0 | 0% | - |
| **2.5 Monitoring of outcome(s) of behaviour without feedback** | 0 | 0% |  | 1 | 3% | health provider |
| **2.7 Feedback on outcome(s) of behaviour** | 13 | 25% | health provider, computer, audiotape | 3 | 9% | health provider |
| 1. **Social support** | | | | | | |
| **3.1 Social support (unspecified)** | 46 | 87% | health provider, video, computer, printed materials, web | 29 | 91% | health provider |
| **3.2 Social support (practical)** | 4 | 8% | Health provider, computer | 1 | 3% | health provider |
| **3.3 Social support (emotional)** | 1 | 2% | Health provider | 0 | 0% | - |
| 1. **Shaping knowledge** | | | | | | |
| **4.1 Instructions on how to perform the behaviour** | 46 | 87% | health provider, video, computer, printed material, web | 22 | 69% | health provider, printed materials |
| **4.2 Information about antecedents** | 1 | 2% | health provider | 0 | 0% | - |
| **4.3 Re-attribution** | 6 | 11% | health provider, video | 0 | 0% | - |
| 1. **Natural consequences** | | | | | | |
| **5.1 Information about health consequences** | 43 | 81% | health provider, video, computer, audiotape, printed material, web | 26 | 81% | health provider, printed materials |
| **5.2 Salience of consequences** | 2 | 4% | health provider, video | 3 | 9% | health provider, video |
| **5.3 Information about social and environmental consequences** | 37 | 70% | health provider, video, web, printed material | 17 | 53% | health provider, printed materials |
| **5.4 Monitoring of emotional consequences** | 9 | 17% | health provider, audiotape | 0 | 0% | - |
| **5.5 Anticipated regret** | 3 | 6% | health provider, video | 0 | 0% | - |
| **5.6 Information about emotional consequences** | 4 | 8% | health provider, video | 1 | 3% | health provider |
| 1. **Comparison of behaviour** | | | | | | |
| **6.1 Demonstration of the behaviour** | 29 | 55% | health provider, video, computer | 6 | 19% | health provider, video |
| **6.2 Social comparison** | 4 | 8% | health provider, video | 2 | 6% | health provider |
| **6.3 Information about others’ approval** | 12 | 23% | health provider, video, computer | 0 | 0% | - |
| 1. **Associations** | | | | | | |
| **7.1 Prompt/cues** | 3 | 6% | health provider, computer | 0 | 0% | - |
| 1. **Repetition and substitution** | | | | | | |
| **8.1 Behavioural practice/rehearsal** | 20 | 38% | health provider | 1 | 3% | health provider |
| **8.7 Graded tasks** | 1 | 2% | health provider | 0 | 0% | - |
| 1. **Comparison of outcomes** | | | | | | |
| **9.1 Credible source** | 49 | 92% | health provider, video, computer, web | 30 | 94% | health provider |
| **9.2 Pros and cons** | 11 | 21% | health provider, video | 1 | 3% | health provider |
| **9.3 Comparative imagining of future outcomes** | 3 | 6% | Video | 0 | 0% | -- |
| 1. **Reward and threat** | | | | | | |
| **10.3 Non-specific reward** | 1 | 2% | health provider | 0 | 0% | - |
| **10.4 Social reward** | 4 | 8% | health provider, computer | 4 | 13% | health provider |
| **10.11 Future punishment** | 1 | 2% | health provider | 1 | 3% | health provider |
| 1. **Regulation** | | | | | | |
| **11.2 Reduce negative emotions** | 2 | 4% | health provider | 0 | 0% | - |
| 1. **Antecedents** | | | | | | |
| **12.2 Restructuring the social environment** | 2 | 4% | health provider | 1 | 3% | health provider |
| **12.3 Avoidance/reducing exposure to cues for the behaviour** | 1 | 2% | health provider, video | 2 | 6% | health provider |
| **12.5 Adding objects to the environment** | 15 | 28% | health provider | 12 | 38% | health provider |
| 1. **Identity** | | | | | | |
| **13.1 Identification of self as role model** | 4 | 8% | health provider, video | 0 | 0% | - |
| **13.2 Framing/reframing** | 10 | 19% | health provider, video | 1 | 3% | health provider |
| **13.3 Incompatible beliefs** | 6 | 11% | health provider, computer | 0 | 0% | - |
| **13.4 Valued self-identify** | 3 | 6% | health provider, computer | 0 | 0% | - |
| 1. **Self-belief** | | | | | | |
| **15.1 Verbal persuasion about capability** | 4 | 8% | health provider, video | 0 | 0% | - |
| 1. **Covert learning** | | | | | | |
| **16.2 Imaginary reward** | 1 | 2% | computer | 0 | 0% | - |
| **16.3 Vicarious consequences** | 3 | 6% | health provider, video | 0 | 0% | - |
| Total BCTs identified | 48 | | | 29 | | |
