# Supplementary material for: Behaviour change techniques in brief interventions to prevent HIV, STI and unintended pregnancies: A systematic review
Source: PLoS One. 2018 Sep 27;13(9):e0204088. doi: 10.1371/journal.pone.0204088 (PMC6159869; doi:10.1371/journal.pone.0204088)
Supplement: S4 Table — (DOCX) [file pone.0204088.s004.docx]

**S4 Table. Summary of the BCTs frequently used in interventions according to duration, intensity, delivery and level of the intervention.**

| **Interventions** | **Effective interventions;** n=53 | | **Control/ineffective interventions;** n=32 | |
| --- | --- | --- | --- | --- |
| **Intensity** | 1 session; n= 46 | 1 + 1 boost session; n=7 | 1 session; n=28 | 1 + 1 boost session; n=4 |
| BCTs | Number of BCTs identified: 42 BCTs; Median BCTs: 13 | Number of BCTs identified: 35 BCTs; Median BCTs:10 | Number of BCTs identified: 28 BCTs; Median BCTs: 6 | Number of BCTs identified: 6 BCTs; Median BCTs: 2.5 |
| BCTs frequently* used | - 1.2 Problem solving (54.3%) - 2.2 Feedback on behaviour (50%) - 3.1 Social support (unspecified) (84.8%) - 4.1 Instructions on how to perform the behaviour (87%) - 5.1 Information about health consequences (84.8%) - 5.3 Information about social and environmental consequences (71.7%) - 6.1 Demonstration of the behaviour (54.3%) - 9.1 Credible source (95.7%) | - Goal setting (behaviour) (71%) - Problem solving (71%) - Action planning (71%) - 1.8 Behavioural contract (57%) - 2.2 Feedback on the behaviour (86%) - 3.1 Social support (unspecified) (100%) - 4.1 Instructions on how to perform the behaviour (86%) - 5.1 Information about health consequences (57%) - 5.3 Information about social and environmental consequences (57%) - 6.1 Demonstration of the behaviour (57%) - 9.1 Credible source (71%) | - 3.1 Social support (unspecified) (89%) - 4.1 Instructions on how to perform the behaviour (75%) - 5.1 Information about health consequences (89%) - 5.3 Information about social and environmental consequences (61%) - 9.1 Credible source (93%) | - 3.1 Social support (unspecified) (100%) - 9.1 Credible source (100%) |
| **Duration** | > 40 min; n=30 | ≤ 40 min; n=23 | ≤ 31 min; n=28 | > 31 min; n=4 |
| BCTs | Number of BCTs identified: 33 BCTs; Median BCTs: 9.5 | Number of BCTs identified: 15 BCTs; Median BCTs: 9 | Number of BCTs identified: 25 BCTs; Median BCTs:5.5 | Number of BCTs identified: 19 BCTs; Median BCTs: 8.5 |
| BCTs frequently* used | - 1.2 Problem solving (70%) - 2.2 Feedback on behaviour (57%) - 3.1 Social support (unspecified) (90%) - 4.1 Instructions on how to perform the behaviour (87%) - 5.1 Information about health consequences (90%) - 5.3 Information about social and environmental consequences (73%) - 6.1 Demonstration of the behaviour (53%) - 9.1 Credible source (100%) | - 2.2 Feedback on behaviour (52%) - 3.1 Social support (unspecified) (83%) - 4.1 Instructions on how to perform the behaviour (87%) - 5.1 Information about health consequences (70%) - 5.3 Information about social and environmental consequences (65%) - 6.1 Demonstration of the behaviour (56%) - 9.1 Credible source (100%) | - 3.1 Social support (unspecified) (89%) - 4.1 Instructions on how to perform the behaviour (64%) - 5.1 Information about health consequences (79%) - 5.3 Information about social and environmental consequences (54%) - 9.1 Credible source (93%) | - 1.2 Problem solving (50%) - 1.3 Goal setting (outcome) (50%) - 2.2 Feedback on behaviour (50%) - 3.1 Social support (unspecified) (100%) - 4.1 Instructions on how to perform the behaviour (100%) - 5.1 Information about health consequences (100%) - 5.3 Information about social and environmental consequences (50%) - 6.1 Demonstration of the behaviour (50%) - 9.1 Credible source (100%) - 10.4 Social reward (50%) |
| **Delivery** | Health provider-delivered; n=30 | Health provider and multimedia-delivered; n=23 | Health provider delivered; n=26^a^ | Health provider and multimedia delivered; n=5 |
| BCTs | Number of BCTs identified: 46 BCTs; Median BCTs: 10 | Number of BCTs identified: 38 BCTs; Median BCTs: 9 | Number of BCTs identified: 23 BCTs; Median BCTs: 5 | Number of BCTs identified: 18 BCTs; Median BCTs 6.5 |
| BCTs frequently* used | - 1.2 Problem solving (50%) - 2.2 Feedback on behaviour (63%) - 3.1 Social support (unspecified) (83%) - 4.1 Instructions on how to perform the behaviour (77%) - 5.1 Information about health consequences (80%) - 5.3 Information about social and environmental consequences (63%) - 9.1 Credible source (100%) | - 1.2 Problem solving (65%) - 3.1 Social support (unspecified) (91%) - 4.1 Instructions on how to perform the behaviour (100%) - 5.1 Information about health consequences (83%) - 5.3 Information about social and environmental consequences (78%) - 6.1 Demonstration of the behaviour (74%) - 9.1 Credible source (83%) | - 3.1 Social support (unspecified) (96%) - 4.1 Instructions on how to perform the behaviour (65%) - 5.1 Information about health consequences (81%) - 9.1 Credible source (92%) | - 3.1 Social support (unspecified) (60%) - 4.1 Instructions on how to perform the behaviour (80%) - 5.1 Information about health consequences (100%) - 5.2 Salience of consequences (60%) - 5.3 Information about social and environmental consequences (80%) - 6.1 Demonstration of the behaviour (60%) - 9.1 Credible source (100%) |
| **Level of the intervention** | Individual level; n=37 | Group level; n=16 | Individual level; n=26 | Group level; n=6 |
| BCTs | Number of BCTs identified: 46 BCTs; Median BCTs: 11 | Number of BCTs identified: 22 BCTs; Median BCTs: 7.5 | Number of BCTs identified: 23 BCTs; Median BCTs: 6 | Number of BCTs identified: 13 BCTs; Median BCTs:4.5 |
| BCTs frequently* used | - 1.2 Problem solving (68%) - 2.2 Feedback on behaviour (65%) - 3.1 Social support (unspecified) (92%) - 4.1 Instructions on how to perform the behaviour (95%) - 5.1 Information about health consequences (86%) - 5.3 Information about social and environmental consequences (62%) - 6.1 Demonstration of the behaviour (57%) - 9.1 Credible source (89%) | - 3.1 Social support (unspecified) (75%) - 4.1 Instructions on how to perform the behaviour (69%) - 5.1 Information about health consequences (69%) - 5.3 Information about social and environmental consequences (88%) - 6.1 Demonstration of the behaviour (50%) - 9.1 Credible source (100%) | - 3.1 Social support (unspecified) (96%) - 4.1 Instructions on how to perform the behaviour (77%) - 5.1 Information about health consequences (96%) - 5.3 Information about social and environmental consequences (54%) - 9.1 Credible source (96%) | - 3.1 Social support (unspecified) (67%) - 5.2 Salience of consequences (50%) - 5.3 Information about social and environmental consequences (50%) - 9.1 Credible source (83%) - 12.5 Adding objects to the environment (50%) |

* identified in at least 50% of the interventions; ^a^ Warner et al. ^[47]^ control/ineffective intervention was printed materials-delivered; BCTS= Behaviour Change Techniques
