# Supplementary material for: Behaviour change techniques in brief interventions to prevent HIV, STI and unintended pregnancies: A systematic review
Source: PLoS One. 2018 Sep 27;13(9):e0204088. doi: 10.1371/journal.pone.0204088 (PMC6159869; doi:10.1371/journal.pone.0204088)
Supplement: S1 File — (DOCX) [file pone.0204088.s005.docx]

**S1 File. Search strategy.**

Published materials: MeSH PubMed search and keyword Summons Search. No Language restriction. Date: 1988-present.

Search strategies for techniques/intervention from other reviews: intervention, interventions, behavior change, review, health behavior, health education, health policy, encourage, impact, increase, decrease, patient education.

Program evaluation or Outcome stud(ies) or Primary Prevention or Impact stu(dies) or Follow-up stud(ies) or Intervention or Education or Preventive/ion or Evaluation/ing or Program or Promotion or Outcome(s) or Initiative or Design.

DRAFTS BSC filter:

Brief: “brief” [TIAB] OR “one-off” [TIAB] OR “one hour” [TIAB] OR “1 hour” [TIAB] OR “fifteen minutes” [TIAB] OR “15 minutes” [TIAB] “60 minutes” [TIAB] “45 minutes” [TIAB] OR “thirty minutes” [TIAB] OR “30 minutes” [TIAB] OR “quick” [TIAB] OR “unique” [TIAB] OR “minutes” [TIAB] OR “limited” [TIAB] OR “short” [TIAB] OR “compressed” [TIAB] OR “succinct” [TIAB] OR “short-term” [TIAB] OR “finite” [TIAB] OR “abrupt” [TIAB] OR “brisk” [TIAB] OR “expeditious” [TIAB] OR “instantaneous” [TIAB] OR “prompt” [TIAB] OR “rapid” [TIAB] OR “sudden” [TIAB] OR “accelerated” [TIAB] OR “fast” OR “high-speed” [TIAB] OR “swift” [TIAB]

Type of Counselling: "face-to-face" [TIAB] OR "Videotape Recording"[Mesh] OR "Videotape Recording" [TIAB] OR "Video Games"[Mesh] OR "Video Games"[TIAB] OR "Video Recording"[Mesh] "Video Recording"[TIAB] OR "Computer Terminals"[Mesh] "Computer Terminals"[TIAB] OR "Webcasts as Topic"[Mesh] OR "Multimedia"[Mesh] OR “Multimedia” [TIAB]

Intervention: (((("Early Medical Intervention"[Mesh]) OR "Intervention Studies"[Mesh]) OR "Evidence-Based Practice"[Mesh]) OR "Controlled Clinical Trials as Topic"[Mesh]) OR "Controlled Clinical Trial" [Publication Type] OR ("Review" [Publication Type]) OR ( "Review Literature as Topic"[Mesh] OR "Peer Review, Health Care"[Mesh] OR "Peer Review, Research"[Mesh] ) OR Health behavior [Mesh] OR "Health Education"[Mesh] OR ("Health Impact Assessment"[Mesh]) OR "Program Evaluation"[Mesh] OR "Patient Education as Topic"[Mesh] OR “program evaluation” (Mesh)

HIV/STI:

Human Immunodeficiency Virus or HIV or Acquired Immunodeficiency Syndrome or AIDS or AIDS or Sexually Transmitted Diseases or STD or STI and prevent(ion/ing)

Sex:

Sex(ual) Behavior(s) or (Sex(ual)) Risk(y) Behavior(s) or Sex(ual) Risk Reduction or Sex(ual) Risk Taking or Sex(ual) Risk Avoidance or Abstinence or Postpon(ing) sex/intercourse or Delay(ing) sex/intercourse or Sexual Activity or Sexual Acts or Protected Sex or Sexual Involvement

Unsafe Sex or ((unsafe or high-risk or unprotected) sex$).tw.

Inclusion/exclusion

Type of Study:

Include: RCTs; Observational; Qualitative (grounded theory, ethnography, descriptive, phenomenological)

Exclude: Discussion papers, commentaries

Population:

Include: individual and group, all ages

Main target: adolescents and young people

Intervention:

Include: Face to face, multimedia, combination face to face and multimedia

Exclude: population level (mass media campaigns)

Comparison:

Include: no intervention/standard care

Exclude: studies without a control

Language:

No Limitations

Date:

1988*-2014

*initial year for published studies on sexual behavioral counseling in the post-HIV era (<http://www.uspreventiveservicestaskforce.org/uspstf08/sti/stiart.htm>)

Outcomes:

Include: Biologic, behavior, at least 3 month follow up (social-psychological outcomes (including attitudes towards condoms or HIV or intentions to use condoms)

Setting:

Inclusion: Primary care settings, Specialty clinics

Exclusion: school based sex education programs

Keywords:

Keywords of BSC: brief therapy, brief communication, attending, empathy, client focus, observation, representation, open questions, active listening, emotional reformulation, motivation, personal goal, helping model, problem solving, empowerment, awareness

Other Keywords: sexual health, reproductive health, primary care, cancer, sexual dysfunction, sexual distress, sexual concerns, sexual difficulties, sexual problems, sexual misconceptions, sexually transmitted infections, HIV, unwanted pregnancy, abortion, sexual violence, harmful practices, knowledge increase, well-being, autonomy, pleasure, training
