# Supplementary material for: Behaviour change techniques in brief interventions to prevent HIV, STI and unintended pregnancies: A systematic review
Source: PLoS One. 2018 Sep 27;13(9):e0204088. doi: 10.1371/journal.pone.0204088 (PMC6159869; doi:10.1371/journal.pone.0204088)
Supplement: S2 File — (DOCX) [file pone.0204088.s006.docx]

**S2 File. List of 93 BCTs and clusters.**

**BCT Taxonomy (v1): 93 hierarchically-clustered techniques (Michie et al.)**

**1 1. Goals and planning**

1.1. Goal setting (behavior)

1.2. Problem solving

1.3. Goal setting (outcome)

1.4. Action planning

1.5. Review behavior goal(s)

1.6. Discrepancy between current behavior and goal

1.7. Review outcome goal(s)

1.8. Behavioral contract

1.9. Commitment

**2. Feedback and monitoring**

2.1. Monitoring of behavior by others without feedback

2.2. Feedback on behaviour

2.3. Self-monitoring of behaviour

2.4. Self-monitoring of outcome(s) of behaviour

2.5. Monitoring of outcome(s) of behavior without feedback

2.6. Biofeedback

2.7. Feedback on outcome(s) of behavior

**3. Social support**

3.1. Social support (unspecified)

3.2. Social support (practical)

3.3. Social support (emotional)

**4. Shaping knowledge**

4.1. Instruction on how to perform the behavior

4.2. Information about antecedents

4.3. Re-attribution

4.4. Behavioral experiments

**5. Natural consequences**

5.1. Information about health consequences

5.2. Salience of consequences

5.3. Information about social and environmental consequences

5.4. Monitoring of emotional consequences

5.5. Anticipated regret

5.6. Information about emotional consequences

**6. Comparison of behaviour**

6.1. Demonstration of the behavior

6.2. Social comparison

6.3. Information about others’ approval

**7. Associations**

7.1. Prompts/cues

7.2. Cue signalling reward

7.3. Reduce prompts/cues

7.4. Remove access to the reward

7.5. Remove aversive stimulus

7.6. Satiation

7.7. Exposure

7.8. Associative learning

**8. Repetition and substitution**

8.1. Behavioral practice/rehearsal

8.2. Behavior substitution

8.3. Habit formation

8.4. Habit reversal

8.5. Overcorrection

8.6. Generalisation of target behavior

8.7. Graded tasks

**9. Comparison of outcomes**

9.1. Credible source

9.2. Pros and cons

9.3. Comparative imagining of future outcomes

**10. Reward and threat**

10.1. Material incentive (behavior)

10.2. Material reward (behavior)

10.3. Non-specific reward

10.4. Social reward

10.5. Social incentive

10.6. Non-specific incentive

10.7. Self-incentive

10.8. Incentive (outcome)

10.9. Self-reward

10.10. Reward (outcome)

10.11. Future punishment

**11. Regulation**

11.1. Pharmacological support

11.2. Reduce negative emotions

11.3. Conserving mental resources

11.4. Paradoxical instructions

12. Antecedents

12.1. Restructuring the physical environment

12.2. Restructuring the social environment

12.3. Avoidance/reducing exposure to cues for the behavior

12.4. Distraction

12.5. Adding objects to the environment

12.6. Body changes

**13. Identity**

13.1. Identification of self as role model

13.2. Framing/reframing

13.3. Incompatible beliefs

13.4. Valued self-identify

13.5. Identity associated with changed behavior

**14. Scheduled consequences**

14.1. Behavior cost

14.2. Punishment

14.3. Remove reward

14.4. Reward approximation

14.5. Rewarding completion

14.6. Situation-specific reward

14.7. Reward incompatible behavior

14.8. Reward alternative behavior

14.9. Reduce reward frequency

14.10. Remove punishment

**15. Self-belief**

15.1. Verbal persuasion about capability

15.2. Mental rehearsal of successful performance

15.3. Focus on past success

15.4. Self-talk

**16. Covert learning**

16.1. Imaginary punishment

16.2. Imaginary reward

16.3. Vicarious consequences
